# State transitions through inhibitory interneurons in a cortical network model

**DOI:** 10.1371/journal.pcbi.1009521

**Published:** 2021-10-15

**Authors:** Alexander Bryson, Samuel F. Berkovic, Steven Petrou, David B. Grayden

**Affiliations:** 1 Ion Channels and Disease Group, The Florey Institute of Neuroscience and Mental Health, University of Melbourne, Australia; 2 Department of Neurology, Austin Health, Heidelberg, Australia; 3 Epilepsy Research Centre, Department of Medicine, University of Melbourne, Austin Health, Heidelberg, Australia; 4 Department of Biomedical Engineering, University of Melbourne, Melbourne, Australia; Université Paris Descartes, Centre National de la Recherche Scientifique, FRANCE

## Abstract

Inhibitory interneurons shape the spiking characteristics and computational properties of cortical networks. Interneuron subtypes can precisely regulate cortical function but the roles of interneuron subtypes for promoting different regimes of cortical activity remains unclear. Therefore, we investigated the impact of fast spiking and non-fast spiking interneuron subtypes on cortical activity using a network model with connectivity and synaptic properties constrained by experimental data. We found that network properties were more sensitive to modulation of the fast spiking population, with reductions of fast spiking excitability generating strong spike correlations and network oscillations. Paradoxically, reduced fast spiking excitability produced a reduction of global excitation-inhibition balance and features of an inhibition stabilised network, in which firing rates were driven by the activity of excitatory neurons within the network. Further analysis revealed that the synaptic interactions and biophysical features associated with fast spiking interneurons, in particular their rapid intrinsic response properties and short synaptic latency, enabled this state transition by enhancing gain within the excitatory population. Therefore, fast spiking interneurons may be uniquely positioned to control the strength of recurrent excitatory connectivity and the transition to an inhibition stabilised regime. Overall, our results suggest that interneuron subtypes can exert selective control over excitatory gain allowing for differential modulation of global network state.

## Author summary

Inhibitory interneurons comprise a significant proportion of all cortical neurons and play a crucial role in sustaining normal neural activity in the brain. Although it is well established that there exist distinct subtypes of interneurons, the impact of different interneuron subtypes upon cortical function remains unclear. In this work, we explore the role of interneuron subtypes for modulating neural activity using a network model containing two of the most common interneuron subtypes. We find that one interneuron subtype, known as fast spiking interneurons, preferentially control the strength of activity between excitatory neurons to regulate changes in network state. These findings suggest that interneuron subtypes may selectively modulate cortical activity to promote different computational capabilities.

## Introduction

Despite comprising only 15 to 20% of cortical neurons, inhibitory interneurons are crucial for the normal operation and computational power of the cortex[1–3]. Inhibitory interneurons maintain a balance of network excitation and inhibition and restrain the emergence of pathological excitatory activity such as epileptic seizures[4,5].

Beyond simply ‘applying the brakes’ to excitation, however, interneurons also support the existence of network states with specific computational capabilities[2,6]. The presence of excitation-inhibition (EI) balance, in which inhibitory (hyperpolarising) synaptic currents are of similar magnitude to excitatory currents, promotes asynchronous neuronal spiking and maximises network entropy[5,7–9]. Conservation of EI ratios through the adjustment of inhibitory synapses has been observed across pyramidal cell populations, and EI balance can also exhibit temporal characteristics, whereby inhibition tracks excitation with short time lag[10–12]. Rapid temporal EI balance may allow for efficient neural coding and provide a method for gating cortical information transfer[11,13]. Finally, powerful connectivity between inhibitory and excitatory neurons can stabilise networks possessing strong excitatory-to-excitatory interactions required for tasks such as short-term memory formation[14,15]. Networks possessing these properties are known as inhibition-stabilised networks (ISN) and are associated with unique computational properties such as non-linear receptive field response summation[16,17]. Cortical networks with features consistent with ISNs have been demonstrated *in-vivo* across multiple cortical regions and during both awake and anaesthetised states [16,18].

A remarkable feature of inhibitory interneurons is their diversity of electrophysiological properties, gene expression patterns and morphology compared to excitatory neurons[19–21]. Two prevalent and well-studied interneuron subtypes include parvalbumin and somatostatin interneurons[3]. Parvalbumin interneurons typically exhibit ‘fast spiking’ (FS) electrophysiological features with narrow action potential width and rapid firing rates, and preferentially synapse onto the peri-somatic region of excitatory pyramidal cells[3,6,22]. In contrast somatostatin interneurons typically exhibit ‘non-fast spiking’ (NFS) features with wider action potential width and slower firing rates, and preferentially synapse onto the dendritic tree of pyramidal cells.

The presence of interneuron populations with distinct properties may allow for interneuron subtype-specific influence over cortical function and EI balance[1,2,6]. For example, the peri-somatic distribution of FS interneuron synapses enables precise regulation of spike timing and correlations between excitatory neurons, and differences in membrane kinetics between FS and NFS interneurons may promote network oscillations at different frequency bands[2]. Although this hypothesis is supported by the observation that the excitability of interneuron subtypes can be selectively modulated through the action of certain neurotransmitters such as acetylcholine, serotonin and GABA, the precise role of interneuron subtypes for regulating cortical activity remains unclear[23–25].

Therefore, we explored the influence of interneuron subtypes over cortical activity using a spiking network model with one population of excitatory pyramidal cells (PC) and two inhibitory neuronal populations: FS and NFS interneurons. The excitability of each interneuron population was modulated by changing neuronal rheobase (or threshold) and evaluating network spike characteristics and EI balance. We found that network properties were sensitive to modulation of the FS population, and that reduced FS excitability produced a paradoxical reduction of EI balance. Using both a rate-based model and further analysis of our spiking network, we found that reduced FS excitability can selectively enhance gain within the PC population and mediate the transition into an inhibition stabilised state. An exploration of the biophysical properties of the FS and NFS interneuron populations revealed that the rapid intrinsic response properties and short synaptic latencies associated with FS interneurons enables preferential modulation of PC population gain.

## Methods

### Overview and rationale of network model

There exists inter-species, regional and laminar variations in the cellular composition and connectivity of cerebral cortex. For instance, the proportion of inhibitory interneurons to excitatory pyramidal cells may range from 9% to 20% across layers of mouse somatosensory cortex, and the proportion of PV to non-PV interneuron subtypes from 30% to 60%[3,26]. Yet despite this heterogeneity, there exist conserved features that suggest some differences represent a `variation on a theme’ from which common principles can be understood[27]. For example, PV and SST interneurons demonstrate connectivity preference with the peri-somatic and dendritic regions of pyramidal cells, respectively, and connection probability between pyramidal cells and inhibitory interneurons is typically greater than recurrent connectivity between pyramidal cells [26,28,29]. For the purposes of this study, we consider PV and SST interneurons equivalent to FS and NFS, respectively.

To explore the role of interneuron subtypes upon network activity we developed a model that retained broad themes of cortical organisation with respect to intrinsic neuron electrophysiological features, the proportion of neuron subtypes, connection probability and synaptic properties (code available at https://osf.io/2sr6y/). We assume that incorporating these themes will provide insight into principles of cortical network activity, while acknowledging that our model is not necessarily representative of a specific cortical layer or brain region.

### Pyramidal cell model

Excitatory pyramidal cell (PC) models possess both a proximal (‘somatic’) and distal (‘dendritic’) compartment and were optimised to exhibit realistic electrophysiological properties in response to constant current stimuli. The soma contained a rapid-activating sodium (NaT), persistent potassium (K_P_), hyperpolarisation-activated (Ih) and leak (L) conductance. The dendrite contained an Ih, leak and high-voltage activated calcium (CaH) conductance, and calcium accumulation/decay kinetics (channel mechanisms of *Markram et al*.[30] and their cited references). The current-balance equations for somatic (*S*) and dendritic (*D*) compartments are

$$C_{S}\frac{dv_{S}}{dt}=G_{NaT}m_{NaT}^{3}h_{NaT}(v-E_{Na})+G_{K_{P}}m_{K_{P}}^{2}h(v-E_{K_{P}})+G_{Ih}m_{Ih}(v-E_{Ih})+G_{L}(V-E_{L})+\frac{v_{D}}{R_{SD}}$$

$$C_{D}\frac{dv_{D}}{dt}=G_{CaH}m_{CaH}^{2}h_{CaH}(v-E_{CaH})+G_{Ih}m_{Ih}(v-E_{Ih})+G_{L}(v-E_{L})+\frac{v_{S}}{R_{SD}}$$ (1)

$$m_{x}=\frac{m_{\infty }(v)-m_{x}}{m_{\tau }(v)}\;h_{x}=\frac{h_{\infty }(v)-h_{x}}{h_{\tau }(v)}$$

Where *C* is the membrane capacitance, *G* the peak channel conductance, *v* the transmembrane voltage, *E* the channel reversal potential, *R*_SD_ the intracellular resistance between somatic and dendritic compartments, *m*/*h* the activation/inactivation gating variables, *m*_∞_(*v*) and *h*_∞_(*v*) the steady-state and time-constant functions, and *x* represents the channel mechanism,. Peak channel conductance, membrane capacitance, intracellular resistance and calcium kinetics were optimised using a feature-based evolutionary algorithm in response to the following external current stimuli based on *Hay et al*.[31,32]:

1. 5ms, 1.9nA, constant current at the soma
2. 5ms, 1.9nA, constant current & 5ms 0.2nA constant current at the dendrite at offset of somatic stimulus
3. 500ms, 0.5nA, constant current at the soma
4. 500ms, 0.8nA, constant current at the soma

Parameters were optimised to reproduce AP waveform, back-propagating AP (BAP), and BAP-activated Ca^2+^ spike features based on *Hay et al*[32]. A list of features and objective values is provided in **S1 Table**. Given that our model contained only two compartments, feature 9 from *Hay et al*. (bAP amplitude at 620um) was excluded and to ensure realistic time lag between APs generated at the soma and dendrite two additional features were added: times to first spike at the soma and dendrite. Finally, to ensure realistic input-spike frequency characteristics, the model was optimised to exhibit spike frequencies of 10 Hz and 20 Hz to stimuli 3 and 4, respectively, similar to the spike frequency generated by the model of *Hay et al*. [32]

### Interneuron & simplified PC model

FS and NFS interneurons, and the simplified PC model, were described using the formulation of *Izhikevich et al*[33,34],

$$\frac{dv}{dt}=0.04v^{2}+fv+g-u+I$$

$$\frac{du}{dt}=a(bv-u)\;if\;v\geq X\;then\;\left\{ \begin{array}{c} v\leftarrow c\\ u\leftarrow u+d \end{array}\right.$$ (2)

Parameter values for FS and NFS interneurons were set to those found in *Izhikevich et al*[33,34] to replicate features of FS and ‘low threshold spiking’ electrophysiologic subtypes, respectively. The simplified PC model (the “Izhikevich PC model”) exhibited ‘regular spiking’ characteristics. To modify interneuron rheobase a constant current (*I*) was applied to the soma. Increased rheobase (reduced excitability) was quantified as the percentage rheobase increase relative to baseline in response to a constant depolarising stimulus.

### Synapse model

Synaptic conductance dynamics were modelled with a bi-exponential formalism,

$$g_{syn}(t)=W\sum_{k}\alpha (t-t_{k})$$ (3)

$$\alpha (t)=F\left(e^{-\frac{t}{\tau_{2}}}-e^{-\frac{t}{\tau_{1}}}\right)$$ (4)

where *t*_*k*_ denotes spike times of presynaptic neurons, *F* is a normalisation factor such that peak conductance equals 1uS and *W* is synaptic weight. Excitatory synapses contain fast (AMPA) and slow (NMDA) conductances with AMPA:NMDA ratio of 0.2, reversal potential of 0mV and τ_1_/τ_2_ set to 0.1/1.5 ms for AMPA and 2/20 ms for NMDA, respectively[35–37]. Rise/decay inhibitory (GABA_A_) conductance time constants were set to 0.25/5 ms with reversal potential of –80 mV.

### Synaptic weights & latencies

A range of synaptic post-synaptic potential (PSP) amplitudes and latencies have been recorded from cortex with significant variation across species and brain regions[3,26,28,38–42]. Nevertheless, there exist broad differences between neuronal populations that we have aimed to capture using a parsimonious approach with the following considerations:

1. PSP amplitudes have consistently been observed to be of greatest magnitude from PC to FS neurons and of smallest magnitude between PC neurons.[26,28,38–41,43]
2. Synaptic latencies have consistently been found to be shortest between PC and FS interneurons and longest between PC neurons.[3,26,28,39,40,42]

Given these observations, synaptic weights were adjusted to generate mean peak PSPs of 0.8 mV for PC-to-FS connections, 0.4 mV for PC-to-PC connections and 0.6 mV for other connections. These values are comparable to recordings from mouse somatosensory cortex[26,28]. The distribution of experimentally measured PSPs are typically skewed and so weights were drawn from a log-normal distribution that generated a PSP sample variance of 0.1 mV[26,39]. To account for filtering of synaptic input, synaptic weights onto the PC dendrite were scaled to generate a PSP amplitude two-thirds of the PSP elicited at the soma, a value that approximates the somatic PSP amplitude in response to synaptic input ~150 um from the soma in the PC model of *Hay et al*. [32]

To reflect population differences in synaptic latencies, mean latencies from PC-to-FS and from PC-to-PC neurons were set to 1.0 ms and 1.8 ms, respectively. Other mean latencies were set to 1.5 ms, values comparable to recordings from mouse somatosensory cortex[26,28,40]. Latencies were drawn from a normal distribution with variance of 0.1 ms[39]. To prevent instantaneous or negative values, PC-to-FS latencies were drawn from a truncated normal distribution with lower limit of 0.1 ms.

### Cell numbers & connectivity

The network contained 1800 (80%) PC neurons and 450 (20%) inhibitory interneurons. These values approximate neuron numbers and excitatory-inhibitory cell ratios within a single cortical column layer[26]. Inhibitory interneurons were divided equally between FS and NFS subtypes, which approximates proportions measured in some studies, although variation exists across layers[3,28,30].

Connection probabilities from 5% to 20% have been estimated between PC neurons within a cortical column. In comparison, connectivity between PC and FS/NFS interneurons has been found to be denser but with a wide range of measured values from 10% to over 50%, which may reflect differences between species, brain region and experimental technique[28,29,40,41,44,45]. A number of studies have found denser connectivity between PC and FS compared to PC and NFS interneurons[28,40]. To reflect these trends, we assigned a connection probability of 15% between PC neurons, 25% between PC and FS interneurons and 20% for all other connections. FS interneurons preferentially synapse onto the axon, soma and proximal dendrites of pyramidal neurons in contrast to NFS interneurons which tend to synapse distally[46,47]. To preserve differences in synaptic distribution we assigned 80% of FS/NFS synapses onto the proximal/distal PC compartment. PC-to-PC synapses were more evenly distributed, with 60% assigned to the soma[48].

### External stimulation & network simulation

Neurons in each population were stimulated with external Poisson-distributed excitatory and inhibitory synaptic stimuli. Excitatory and inhibitory stimulation frequencies and conductance magnitude for each population were equal to generate `balanced’ external input[49]. Cortical excitatory neurons typically fire sparsely compared to inhibitory interneurons, with mean rates often under 1Hz[50–52]. To provide a baseline from which to assess the relative impact of FS and NFS excitability upon network activity, a grid parameter search of external stimulation frequencies upon each population was performed to elicit equal mean firing rates within the FS and NFS populations of 2.0 Hz, and 0.3 Hz within the PC population. An identical approach was used to generate baseline firing frequencies for the network receiving only excitatory external stimulation. Network simulations were performed with NetPyNE using a fixed integration time step (dt = 0.025ms)[53]. Mean values of network properties were obtained by performing 10 simulations initiated with a different random seed.

### One & two-population networks

To isolate the role of interneuron properties and external input upon PC gain, we developed a two-population network with PC and FS interneurons (the “PC-FS network”), and one-population networks with PC, FS and NFS neurons alone (the “FS interneuron network” and “NFS interneuron network”). FS neurons comprised 20% of the population in the PC-FS network (450). Otherwise, cell numbers were identical to the original network. Each network was stimulated by excitatory and inhibitory synapses to elicit the same firing rates as the three-population network under baseline conditions.

### Network spike characteristics & excitation-inhibition balance

Inter-spike interval coefficient of variation (ISICV) for neuron *n* was defined as the ratio of ISI mean (〈ISI_*n*_〉) and ISI standard deviation (*σ*_ISI,n_),

$${ISICV}_{n}=\frac{\langle {ISI}_{n}\rangle }{\sigma_{ISI,n}}$$ (5)

The spike correlation coefficient (CC) was estimated by converting spike trains for each PC neuron into 10 ms binned spike count vectors (***x***) and calculating the mean cross-correlation between all distinct neuron pairs (*a*, *b*) within the PC population (size *N*),

$$CC_{p}=\frac{1}{\left(\begin{array}{c} N\\ 2 \end{array}\right)}\sum_{a=1}^{N-1}\sum_{b=a+1}^{N}\frac{Cov(x_{a},x_{b})}{\sqrt{Var(x_{a})Var(x_{b})}}$$ (6)

Values for mean and standard deviation were calculated across ten simulations initiated with a different random seed for external stimulation. To estimate population synchrony, a Fourier transform was performed on the vector of mean population firing rates binned into 10 ms intervals. Results shown are normalised to the peak value at baseline.

The mean conductance from synaptic inputs from population P onto PC neuron n (${\bar{g}}_{n}^{P}$) was calculated as the temporal average of the vector of mean synaptic input conductance onto neuron n ($\langle {\bar{g}}_{n}^{P}\rangle$),

$${\bar{g}}_{n}^{P}=\langle {\bar{g}}_{n}^{P}\rangle ,\;{\bar{g}}_{n}^{P}=\frac{1}{S_{n,P}}\sum_{i=1}^{S_{n,P}}g_{n,i}^{P}$$ (7)

where *i* represents individual synaptic inputs from population *P* and *S*_*n*,*P*_ is the total number of synapses onto neuron n from population P. P represents either the PC, FS or NFS populations, or input from external stimulation sources. To calculate temporal EI balance the normalised cross-correlation function of the vector of mean conductance onto a PC neuron n from PC (${\bar{g}}_{n,t}^{PC}$) and either FS or NFS (${\bar{g}}_{n,t}^{I}$) synaptic inputs was first computed,

$${EI(k)}_{n}=\frac{\frac{1}{T}\sum_{t=0}^{T-k}\left({\bar{g}}_{n,t}^{PC}-\langle {\bar{g}}_{n}^{PC}\rangle )({\bar{g}}_{n,t+k}^{I}-\langle {\bar{g}}_{n}^{I}\rangle \right)}{\sigma_{g,PC}\sigma_{g,I}}$$ (8)

where *T* is simulation length, *k* is temporal lag, *I* is the inhibitory (FS or NFS) population, $\langle {\bar{g}}_{n}^{PC}\rangle$ is the temporal average of the vector of mean synaptic input conductance from the PC population onto PC neuron *n* and *σ*_*g*_ is the standard deviation of the vector of mean synaptic input conductance onto PC neuron *n*. To estimate average temporal balance ($\bar{EI}(k)$), 500 PC neurons were selected at random and the mean cross-correlation function of all neurons in this sample was calculated,

$$\bar{EI}(k)=\frac{1}{500}\sum_{n=1}^{500}EI{\left(k\right)}_{n}$$ (9)

Temporal EI balance between PC and either the FS or NFS population was calculated using the same approach.

### Assessment of ISN properties & PC gain

In networks containing multiple inhibitory populations, the existence of an ISN can be evaluated by stimulating the inhibitory population and observing a paradoxical reduction of inhibitory current onto the excitatory population in the presence of lower excitatory firing rates [54]. Therefore, we tested for an ISN by applying constant-current stimuli of increasing magnitude to the FS and NFS populations and calculating the change in PC firing rate and inhibitory input current. A stimulus range that elicited up to a 1Hz change in FS and NFS firing rates was used.

To estimate effective synaptic weight of the PC subnetwork, a 1 ms constant current stimulus of 0.9 nA was applied to 20% of PC neurons. An average population response was obtained by applying the stimulus 100 times and calculating the mean spike frequency of the PC population. The stimulus produced a bi-modal response: the first peak from spikes elicited by the stimulus and the second peak via PC-to-PC interactions. To estimate effective synaptic weight, the ratio of spikes recruited via PC-to-PC interactions (P_2_) to spikes elicited by the stimulus (P_1_) was calculated. Periods corresponding to P_1_ and P_2_ were obtained from the bi-modal average population response. P_1_ was defined from stimulus onset to the minimum between peaks. P_2_ was defined from the minimum between peaks to the time at which the average population response returned to this value.

To explore the role of correlated inputs upon PC neuron excitability, artificial spike trains of increasing pairwise spike cross-correlation were generated. Spike correlations were introduced by first creating 100 independent Poisson spike trains of fixed rate, and then shifting randomly selected spikes within a train to fall within 5ms of a spike within a different train. This process was repeated for spike trains of different mean rate.

### Optimisation of firing rate model

A three-population Wilson-Cowan rate model was developed to explore changes in network dynamics associated with increased FS and NFS rheobase,

$$\tau_{E}\frac{dR_{E}}{dt}={-R}_{E}+B_{E}(W_{EE}R_{E}-W_{EF}R_{F}-W_{EN}R_{N}+I_{E})$$

$$\tau_{F}\frac{dR_{F}}{dt}={-R}_{F}+B_{F}(W_{FE}R_{E}-W_{FF}R_{F}-W_{FN}R_{N}+I_{F})$$ (10)

$$\tau_{N}\frac{dR_{N}}{dt}={-R}_{N}+B_{N}(W_{NE}R_{E}-W_{NF}R_{F}-W_{NN}R_{N}+I_{N})$$

where *R*_*x*_ are the mean firing rates of the PC (*x* = E), FS (*x* = F) or NFS (*x = N*) populations, *W*_*yx*_ are the synaptic weights from population *x* to *y*, *τ*_*X*_ time constant of the population firing rate responses, *B*_*x*_ are the population gains and *I*_*x*_ are external stimulation currents. Under the baseline condition, gains for all populations were set to 1.

The synaptic weights of the rate model were optimised to reproduce the firing rates of the spiking network under baseline conditions using the following approach. First, to reflect features of the spiking network, inhibitory synaptic weights onto each population were expressed as a proportion of the integral of the PSP elicited by an excitatory synapse. For example, the FS-to-PC weight is given by,

$$W_{EF}=\frac{\int \left|{PSP}_{EF}\right|}{\int \left|{PSP}_{EE}\right|}W_{EE}$$ (11)

where *PSP*_*EF*_ is the post-synaptic potential elicited by an FS synapse onto a PC neuron at the resting membrane potential. This approach was used to calculate *W*_*EN*_ in terms of *W*_*EE*_, and weights received by the FS & NFS populations in terms of *W*_*FE*_ & *W*_*NE*_, respectively.

Second, to estimate external stimulation (*I*_*x*_), the spiking network was simulated under baseline conditions with weights of internal network connections set to zero. *I*_*E*_, *I*_*F*_ and *I*_*N*_ were assigned mean firing rates of the PC, FS and NFS populations, respectively. Since this approach will contain error related to changes in membrane conductance after removing internal connections, both external stimulation (*I*_*x*_) and free weight parameters (*W*_*EE*_, *W*_*FE*_ & *W*_*NE*_) were optimised using a least-squares algorithm and an iterative approach so that firing rates of the rate model approximated firing rates of the spiking network. First, synaptic weights were optimised so that the rate model firing rates approximated baseline firing rates of the spiking network for fixed *I*_*x*_. *I*_*x*_ bounds were then relaxed by 10% of their original value, synaptic weights fixed to their updated value, and the optimisation repeated. These steps were repeated until the least-squares error was under 0.001. Final parameter values were: *W*_EE_ = 0.95, *W*_FE_ = 9.36, *W*_NE_ = 9.71, *I*_E_ = 0.89, *I*_F_ = 1.44, *I*_N_ = 2.5.

### Estimation of gain with modulation of FS & NFS excitability

To estimate the impact of reductions in FS and NFS excitability upon network dynamics, we assume that synaptic weights and external stimulation remain fixed at baseline values, but the gain (*B*_*x*_) of each population varies with modulation of interneuron excitability. Therefore, to determine excitatory population gain corresponding reductions of FS excitability in the spiking network, *B*_E_ was re-calculated using the optimised synaptic weights and the steady-state firing rates of the spiking network,

$$B_{E}=\frac{R_{E}}{W_{EE}R_{E}-W_{EF}R_{F}-W_{EN}R_{N}+I_{E}}$$ (12)

The same approach was used to calculate *B*_E_ associated with reductions of NFS excitability.

To create a rate model that approximates conditions of reduced FS excitability, PC gain (*B*_E_) was expressed as a function of the total input current onto the PC population (*I*_*T*_) and fitted with a sigmoidal function,

$$B_{E}=f(I_{T})\;I_{T}=W_{EE}R_{E}-W_{EF}R_{F}-W_{EN}R_{N}+I_{E}$$ (13)

Therefore, *B*_E_ approximates PC population gain as total input current increases due to reductions of FS excitability. This approach was also used to estimate *B*_E_ under conditions of reduced NFS excitability to yield two rate models: one for reductions of FS and one for reductions of NFS excitability. Phase portraits and bifurcation analyses were performed using XPPAUT[55].

### Statistics

Results and error bars shown in Figures represent mean ± s.e.m. Comparisons of spiking characteristics, EI balance and changes in PC gain for a given FS/NFS rheobase value were performed using Welch’s *t* test. Changes in PC gain and population characteristics compared to baseline across different rheobase values were performed with one-way ANOVA and post-hoc Tukey’s test. Differences were considered significant if *P* < 0.01.

## Results

### Network model

**Fig 1:**
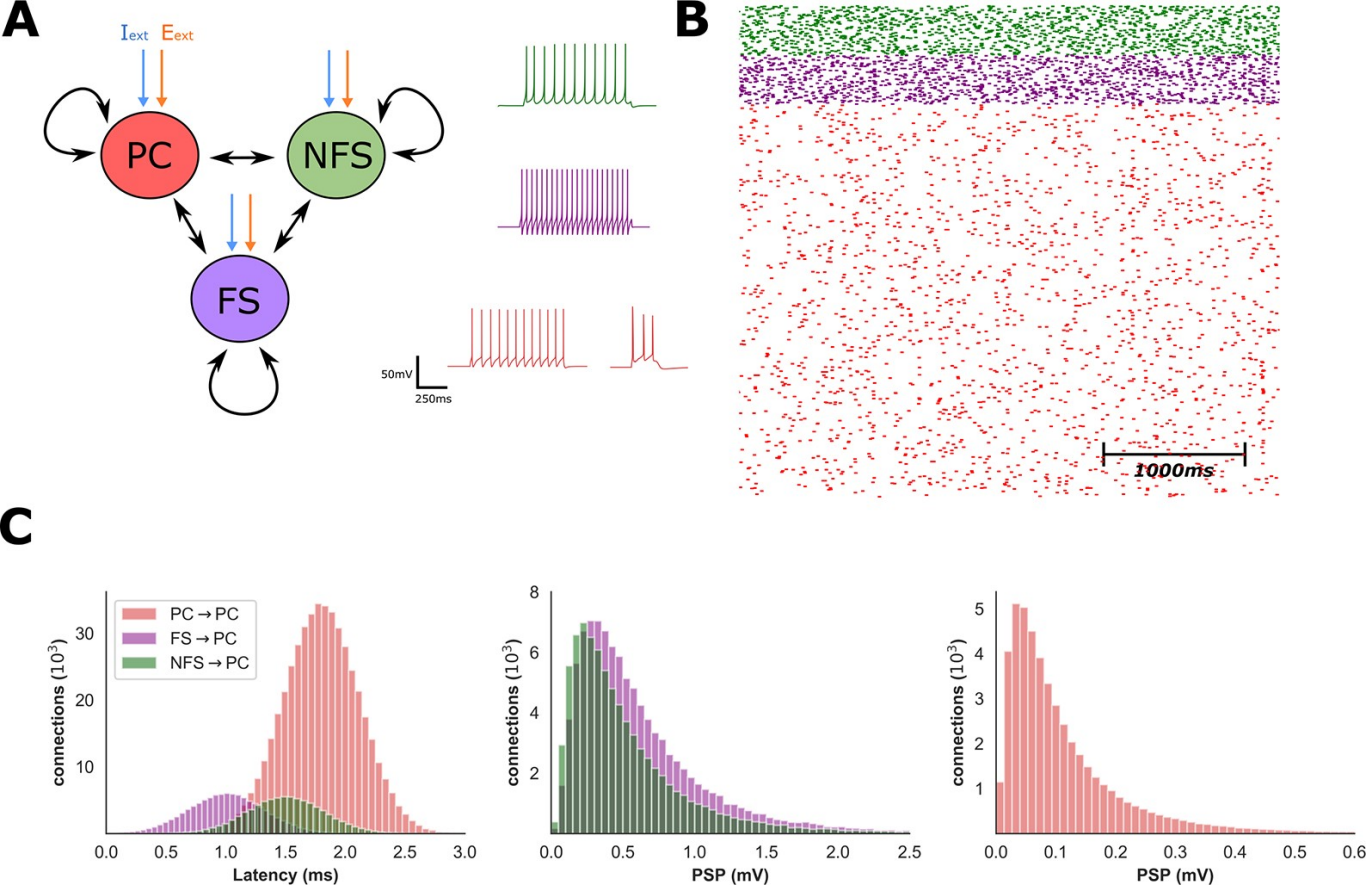
Characteristics of network model. **A)** The network contained one excitatory (PC) and two inhibitory (FS and NFS) populations stimulated by external (E_ext_ and I_ext_) input. Neuron models possessed intrinsic electrophysiological properties typical for each population and, under the baseline conditions, both the FS and NFS populations had mean firing rates of ~2Hz. **B)** Raster plot of network activity under baseline conditions. **C)** Distributions of synaptic latencies and post-synaptic potentials for all synaptic connections onto the PC population. FS to PC connections had shorter latencies and elicited greater post-synaptic potential amplitudes compared with NFS to PC connections.

We developed a network model that incorporated connectivity and synaptic parameters that reflect broad differences between FS and NFS interneuron populations observed in experimental data (**Fig 1A** and **1C**). Notably, connection probability was highest between the FS and PC populations and lowest within the PC population, and synaptic latencies shortest between the FS and PC populations (**Fig 1**). We used a network state in which mean firing rates of the FS and NFS populations are equal (2Hz) as a baseline condition from which to evaluate the impact of interneuron excitability upon network activity.

### Emergent network properties: Spike characteristics & EI balance

A remarkable feature of cortical networks is their ability to maintain a precise balance of excitation and inhibition under different behavioural conditions[12,56,57]. Certain characteristics of EI balance, such as the net current (i.e., the sum of excitatory and inhibitory currents) and temporal relationship determine cortical spiking characteristics and computational properties[58]. Therefore, we evaluated the spiking characteristics and EI balance of our network during the baseline condition to provide a point of comparison with experimental studies.

**Fig 2:**
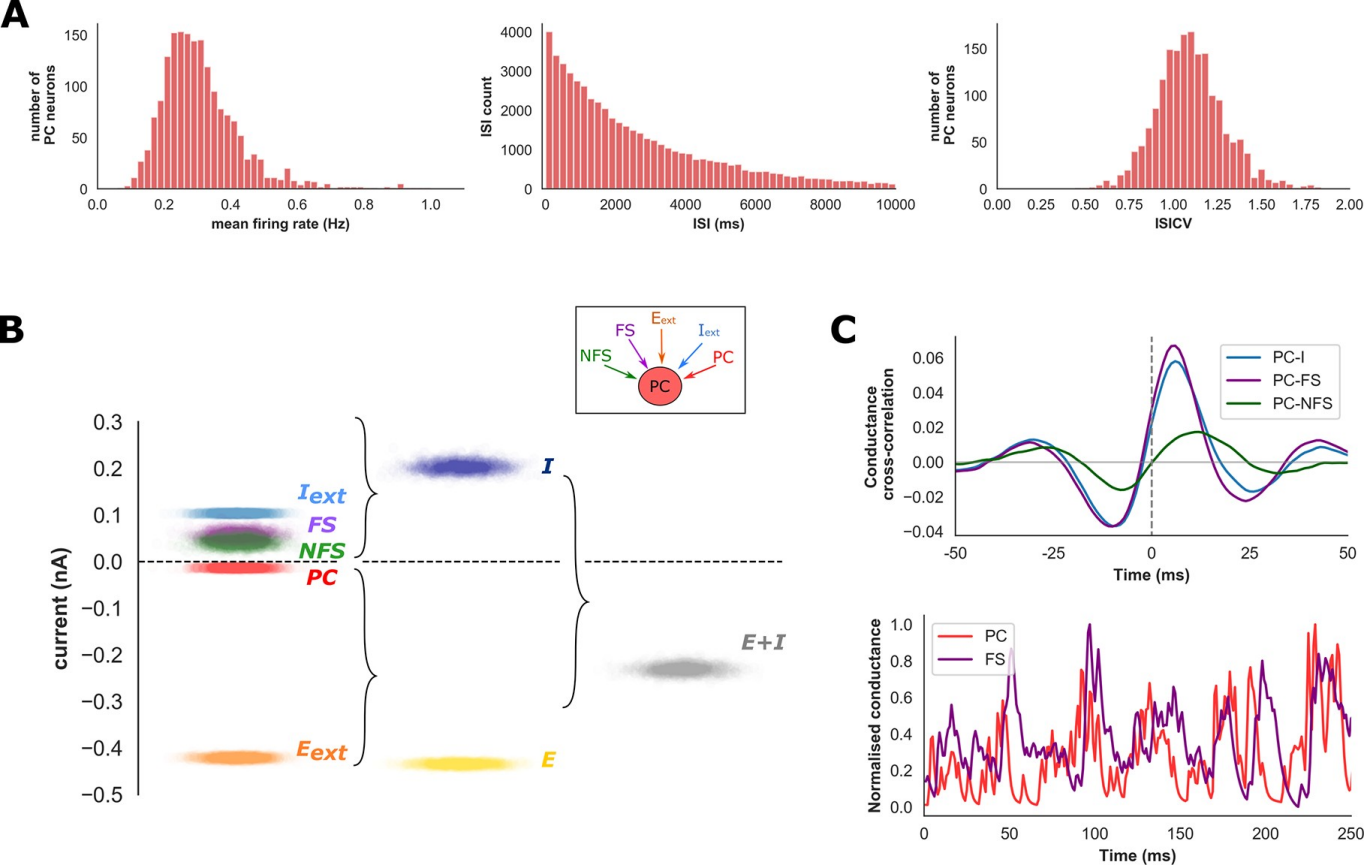
Network spike characteristics and excitation-inhibition balance. **A)** Distributions of spike characteristics for PC neurons under baseline conditions. PC spiking is irregular with a long-tailed ISI distribution and mean ISICV of 1.1. **B)** Breakdown of excitation-inhibition balance and synaptic input current sources onto the PC population during the baseline condition. Points within each cloud represent mean input current from different sources onto individual PC neurons. Mean current from external stimulation (E_ext_, shown in orange) represented the majority (97%) of mean total excitatory current (E, shown in yellow). A similar proportion of inhibitory current was derived from both external (I_ext_) and network sources (FS & NFS). Net current (E+I) was of similar magnitude to total excitatory and inhibitory currents (denoted E & I, respectively). Net conductance onto the PC population (**S1 Fig**) was dominated by inhibitory inputs. **C)** Mean cross-correlation between synaptic conductance’s received from the PC and FS (PC-FS), NFS (PC-NFS) or both inhibitory populations (PC-I) onto the PC population. There is stronger and more rapid cross-correlation between the PC and FS, compared to PC and NFS, populations (peak cross-correlation at 6 and 12ms, respectively). The temporal lag and co-fluctuation of PC and FS synaptic conductance’s can be appreciated from a trace of mean synaptic conductance onto the PC population (**C**, lower).

Under the baseline condition, spiking was highly irregular with a mean ISI coefficient of variation (ISICV) of 1.1 and a long-tailed distribution of inter-spike intervals, qualitatively similar to *in-vivo* observations (**Fig 2**, spike raster plot shown in **Fig 1**)[59,60]. The mean firing rate of most PC neurons (77%) was under 0.5 Hz with a range from 0 to 1.9 Hz and spike correlations within the PC population were very small (0.001) consistent with an irregular and asynchronous network regime[8,61].

To quantify EI balance, we recorded mean synaptic currents and conductance from excitatory and inhibitory inputs onto each PC neurons. Most excitatory current was obtained via external stimulation, and only 3% of the mean total excitatory current onto the entire PC population was obtained via internal network activity from other PC neurons (**Fig 2**). In contrast, 51% of mean total inhibitory synaptic current was obtained from the FS and NFS populations (32% and 19%, respectively). Since FS and NFS populations fire at similar rates under baseline conditions, greater current from the FS population is due to denser connectivity from FS to PC neurons. Finally, mean net current was of similar magnitude to total excitatory current (-0.23 nA compared to -0.43 and 0.2 nA, respectively, **Fig 2**) and net conductance was inhibition-dominated with an EI conductance ratio of 0.5 (**S1 Fig**). The presence of inhibition-dominated synaptic input onto the PC population is consistent with previous intracellular cortical recordings, and the magnitude of net current onto the PC population suggests a ‘loosely balanced’ network regime[49,58]. Similar EI characteristics were observed within the FS and NFS populations (**S1 Fig**).

Intracellular recordings from cortical pyramidal cells have revealed the existence of fast temporal correlations between excitatory and inhibitory synaptic currents, suggesting inhibitory neurons can be rapidly recruited by moment-to-moment fluctuations of excitatory activity[10]. Temporal EI balance may play an important role in maintaining asynchronous activity within densely connected cortical networks[7]. We investigated temporal EI balance characteristics by calculating the mean cross-correlation between network-driven excitatory and inhibitory synaptic conductances onto the PC population. We observed a peak in excitatory-inhibitory cross-correlation onto the PC population at a short time lag of 6ms, consistent with rapid activation of the inhibitory population in response to PC spiking (**Fig 2**). To dissect the relative contributions of FS and NFS interneurons to temporal EI balance, we repeated this analysis for synaptic conductances from each interneuron population. Stronger correlation between PC to FS compared with PC to NFS conductance was observed (0.07 and 0.019, respectively), and peak PC to FS correlations occurred at a shorter time lag compared to PC to NFS (6 ms and 12 ms respectively, **Fig 2**). Correlated excitatory-inhibitory synaptic input is appreciated visually with a trace of normalised mean excitatory and inhibitory conductance onto the PC population (**Fig 2**). Our model, therefore, exhibits rapid recruitment of inhibition in response to fluctuations of excitatory activity, with FS neurons providing faster and more robust inhibitory feedback.

### Modulation of network activity through FS and NFS interneurons

**Fig 3:**
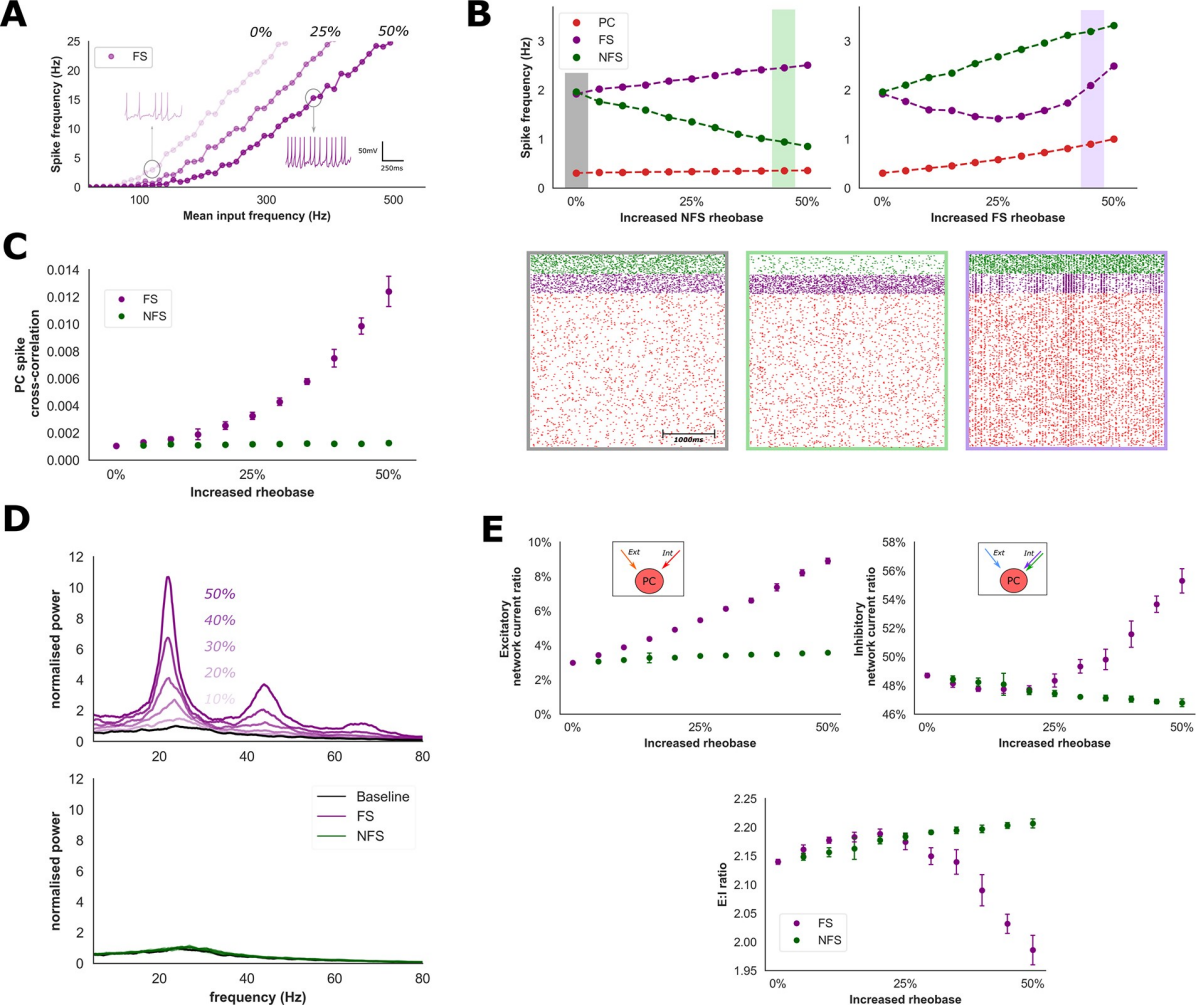
Changes in network properties with modulation of interneuron excitability. **A)** FS interneuron input-frequency relationship following modulation of interneuron excitability in response to random excitatory synaptic input. Interneuron excitability was quantified as the percentage increase in rheobase relative to baseline in response to a constant current stimulus (shown here for baseline, 25% and 50% increase in rheobase). **B)** Increased NFS rheobase produced stepwise reductions of NFS firing rates and increases of FS & PC firing rates. Increased NFS rheobase did not produce a significant change in PC spike correlations (**C**) or population oscillations (**D**). In contrast, an increase in FS firing rates beyond a 25% increase in FS rheobase was observed (**B**, right), together with large increases in both PC spike correlations and population synchrony (normalised relative to baseline, **C** & **D**). **E**) Changes in characteristics of excitatory and inhibitory input current onto the PC population with modulation of interneuron excitability. As FS rheobase was increased, the proportion of excitatory current onto the PC population derived from within the network (i.e., from other PC neurons, denoted Int) increased from ~3 to 9% (**E**, upper left). Since external stimulation remained fixed, this change in network-derived excitatory current must drive increased firing rates observed in **B**. Furthermore, increased FS rheobase also produced greater network-derived inhibition (**E**, top right). Despite increased population firing rates, a paradoxical reduction of EI balance was observed (**E**, bottom).

There is accumulating evidence from studies employing targeted optogenetic silencing or activation that interneuron subtypes may exert selective influence over cortical network state[62–65]. This idea is supported by the observation that some neurotransmitters selectively modulate the excitability of interneuron subtypes[23–25]. Therefore, we explored the impact of reductions of FS and NFS excitability upon network spike characteristics and EI balance. Interneuron excitability was modulated by increasing the rheobase (or spiking threshold) of neurons within the FS and NFS populations (**Fig 3**).

Increasing NFS rheobase reduced NFS firing rates, as would intuitively be expected, and increased PC and FS firing rates consistent with disinhibition from the NFS population (**Fig 3**). As NFS firing rates approach zero PC and FS firing rates plateaued at ~0.4 Hz and ~3.2 Hz, respectively (**S1 Fig**). These changes suggest that the PC population exerts only small influence over NFS firing rates, relative to external excitatory stimulation, because NFS firing rates drop despite higher PC firing rates and excitatory input. Furthermore, the FS population appears to adopt a compensatory role to provide sufficient inhibition to maintain an upper limit of PC firing rates. Finally, a significant change in PC spike correlations and population synchrony was not observed under these conditions (**Fig 3**).

Increased FS rheobase produced a qualitatively different influence upon network activity. Here, the FS population firing rate exhibited a U-shaped relationship when rheobase was increased beyond 25% (**Fig 3**). PC and NFS firing rates also increased. This transition in network activity was associated with a significant and greater than 10-fold increase in both spike correlations (from 0.001 to 0.012, *P* < 0.001 for FS rheobase ≥ 10%, Welch’s *t* test, **Fig 3**) and oscillatory power between 20 and 25Hz within the PC population (*P* < 0.001 for FS rheobase ≥ 10%, Welch’s *t* test, **Fig 3**). Stronger temporal EI correlation between excitatory and inhibitory synaptic inputs were also observed (**S1 Fig**). Interestingly, despite higher firing rates within the PC population, EI balance within the network transitioned to a more inhibitory state with a reduction of the EI ratio and greater recruitment of network-driven inhibitory activity (**Fig 3**). Since external stimulation was fixed across all conditions, increased FS firing rates are therefore driven by increased internal excitatory network activity via the PC population (**Fig 3**). Likewise, an increase in PC firing rates despite higher inhibitory interneuron firing rates implies the existence of enhanced recurrent excitation within the PC population.

### Fast spiking interneurons promote the transition into an inhibition-stabilised state

**Fig 4:**
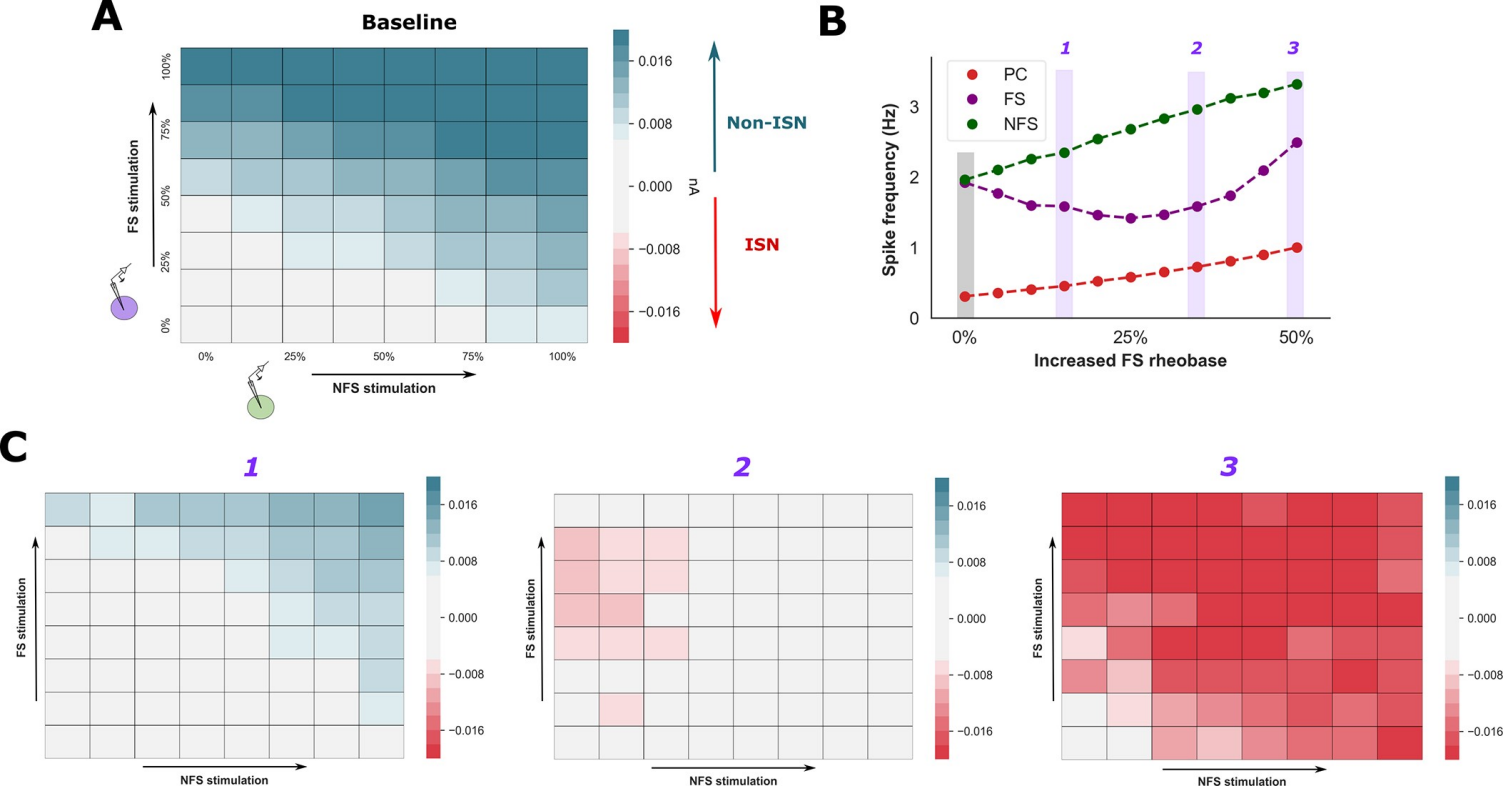
Reduced FS excitability promotes a paradoxical response to inhibitory stimulation. **A)** The presence of an ISN was explored by applying an external current stimulus to the FS (y axis) and NFS (x axis) population and recording the mean change in inhibitory (hyperpolarising) current received by the PC population (increased inhibitory current in green, decreased inhibitory current in red). This process was repeated after increasing FS (**B** & **C**) and NFS (**S2 Fig**) rheobase. During the baseline condition and increased NFS rheobase, external stimulation of the inhibitory population increased inhibitory current received by the PC population. In contrast, increased FS rheobase (**C**) was associated with a paradoxical reduction of inhibitory current received by the PC population despite lower PC firing rates, consistent with an ISN regime. This effect was more pronounced with greater increases of FS rheobase (panel 3).

The presence of strong coupling between excitatory neurons can endow cortical networks with unique computational properties, such as input amplification and pattern completion associated with memory formation, but are also at risk of developing pathologic excitatory activity unless stabilised by recurrent inhibition[15]. Networks with these properties, known as inhibition-stabilised networks (ISN), have been confirmed experimentally by their paradoxical response to external stimulation of the inhibitory population: a reduction of steady-state firing rate in both the inhibitory and excitatory populations[16,66]. After increasing the rheobase of the FS population, we observed changes in network activity to suggest strong coupling between PC neurons and hypothesised that these changes may represent the transition into an ISN. Therefore, we tested for the presence of an ISN by applying an external stimulus to both FS and NFS populations (**Figs 4** and **S2**). Since our network contains two inhibitory populations an ISN is characterised by a reduction in the magnitude of mean inhibitory current onto the PC population despite the presence of lower PC firing rates[54].

Under the baseline condition, external stimulation of the FS and NFS populations enhanced net inhibitory current onto the PC population and reduced PC firing rates, consistent with a non-ISN (**Fig 4**). These changes suggest that greater activity within the inhibitory population exerts a direct inhibitory influence over the PC population, and that reductions of PC firing rate have little bearing over the recruitment of inhibitory neurons. We observed similar findings under conditions of reduced NFS excitability, suggesting that modulation of NFS excitability does not alter the network state (**S2 Fig**).

With reductions of FS excitability under 25%, external stimulation of the inhibitory population also revealed changes consistent with a non-ISN (**Fig 4B** and **4C**). However, further suppression of excitability produced a paradoxical reduction in the magnitude of inhibitory current onto the PC population despite lower PC firing rates, consistent with an ISN (**Fig 4**). Since more external current is applied to the inhibitory population, this ‘reduction of inhibition’ implies diminished recruitment of feedback inhibition driven by the PC population. A more pronounced paradoxical response was observed with greater increases of FS rheobase.

Overall, these results show that interneuron subtypes exert differential control over network state in our model. Notably, reduced FS excitability promoted a regime in which firing rates are driven by PC neurons and are associated with stronger spike correlations and oscillatory activity. To test the generality of these findings, we investigated if network modulation through FS and NFS interneurons is dependent upon either the characteristics of external stimulation applied to the network, or the properties of the model used for the pyramidal cell population. Therefore, we stimulated our network with just excitatory inputs (as opposed to balanced inputs) to reproduce the baseline firing rates, and also developed a second network that contained a simplified single-compartment pyramidal cell model, similar to that used for the FS and NFS populations (the “Izhikevich network”, **S3** and **S4 Figs**). We observed qualitatively similar changes to the original network under both conditions: increasing FS rheobase produced an unexpected reduction of EI balance, the emergence of population oscillations, and promoted a more pronounced paradoxical response to stimulation of the inhibitory populations (**S4 Fig**). Interestingly, one distinguishing feature in the Izhikevich network is the absence of strong spike correlations associated with increased FS rheobase. This difference in considered further in the Discussion.

### Fast spiking interneurons modulate excitatory gain in a rate-based model

**Fig 5:**
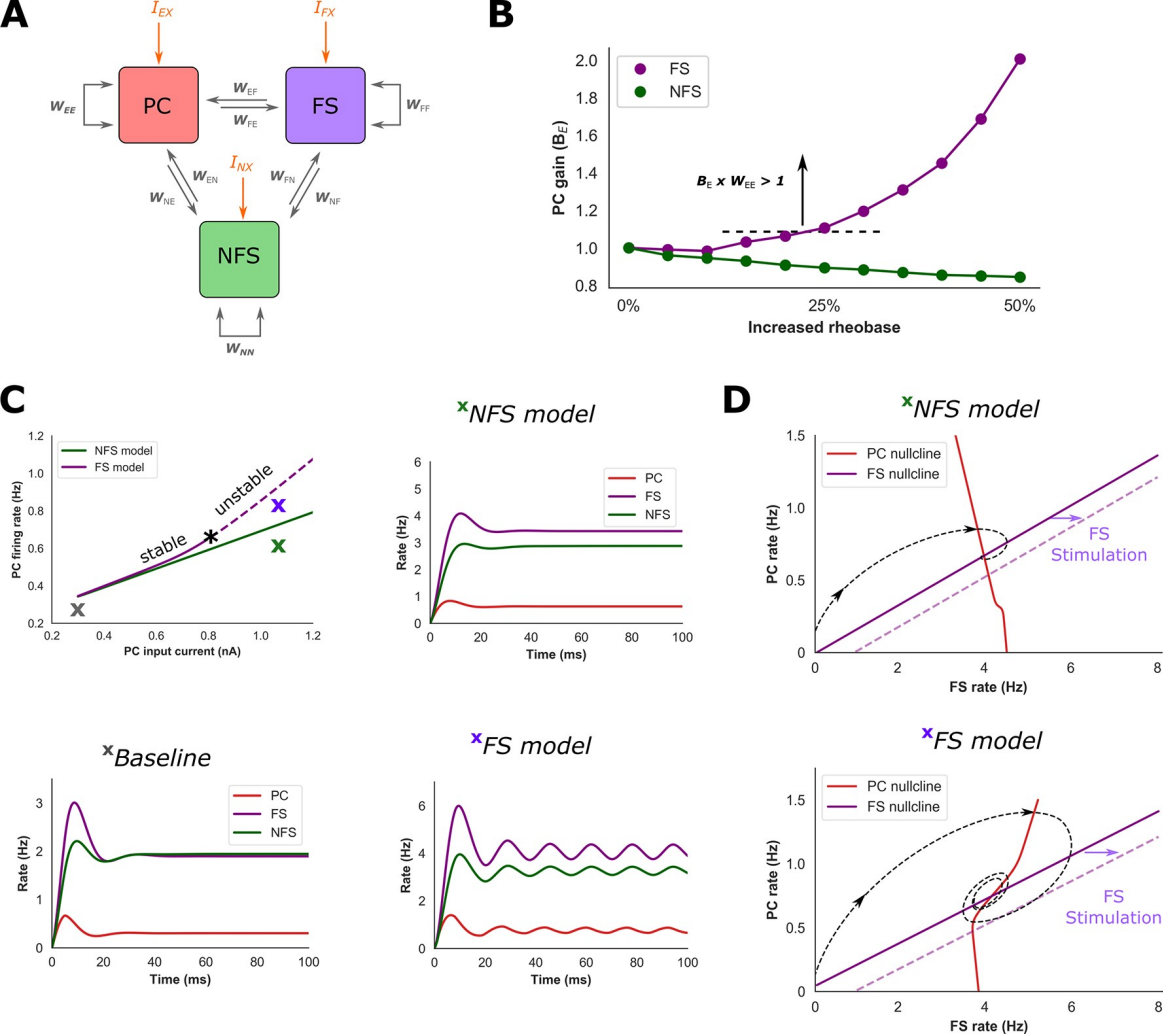
FS interneurons enhance PC gain and promote transition to an ISN regime in a rate model. **A**) A rate model was optimised with synaptic weights (W) based on features of the spiking network. During baseline conditions the gain of each population was set to 1.0. **B**) Under the assumption that synaptic weights remain fixed, changes of PC population gain (B_E_) in the rate model were required to reproduce firing rates from the spiking network associated with increased FS/NFS rheobase. Increased FS rheobase increased B_E_ such that the effective recurrent excitatory synaptic strength (B_E_ x W_EE_) exceeded 1.0. **C)** Two rate models were developed based on changes in PC gain shown in **B**: one approximating increased NFS rheobase (‘NFS model’) and one FS rheobase (‘FS model’, firing rates during baseline conditions in lower left caption). **C**) The FS model loses stability via a Hopf bifurcation as B_E_ x W_EE_ exceeds 1.0 (asterix) and exhibits sustained oscillations, whereas a qualitative change in network behaviour does not occur in the NFS model. **D**) Phase portraits and firing rate trajectories of the NFS and FS models for inputs shown in **C** under the condition of fixed NFS firing rate. The PC nullcline of the FS model has a rightward inflection, and stimulation of the FS population moves the fixed point to lower PC and FS firing rates, suggestive of an ISN regime. In contrast, stimulation of the FS population in the NFS model increases the steady-state FS firing rate, suggestive of a non-ISN regime. These findings are confirmed by changes of inhibitory current onto the PC population in response to stimulation of the FS and NFS population (**S5 Fig**).

Our network model suggests that FS interneurons can preferentially mediate a transition of network activity into an inhibition-stabilised state. To obtain further insight into the dynamics of this transition, we developed a Wilson-Cowan firing rate model with synaptic weights estimated from features of the detailed network (**Fig 5**). The rate model was first optimised to reproduce firing rates of the detailed network during the baseline condition with the gain of each population (*B*_*X*_) set to 1. Under the assumption that synaptic weights are fixed, the firing rates during reduced FS and NFS excitability were then reproduced by re-fitting the gain of each population. This resulted in two distinct models that approximate conditions of reduced FS and NFS excitability, respectively.

To reproduce the baseline firing rates of the detailed model, the PC-to-PC synaptic weight (*W*_*EE*_) was optimised to a value of 0.95 and firing rates settled to a stable fixed point (**Fig 5**). Networks in which the effective excitatory-to-excitatory synaptic weight (*B*_*E*_*W*_*EE*_) is greater than 1 can generate self-sustained excitatory activity required for an ISN regime[15]. Consequently, although at baseline the rate model did not behave as an ISN (confirmed by the response of the model to external stimulation of the inhibitory population, **S5 Fig**), it is possible a small increase in PC gain could reproduce this condition.

To reproduce firing rates corresponding to increased NFS rheobase within the detailed model, the gain of the PC population dropped below 1 (**Fig 5**). Stimulation of the PC population within the NFS model, which approximates conditions of increased NFS rheobase, did not produce a qualitative change in network behaviour with firing rates again reaching a stable fixed point (**Fig 5**). Furthermore, consideration of both the FS-PC phase portrait (**Fig 5C** and **5D**) and the response to external stimulation of the inhibitory population reveal features consistent with a non-ISN regime (**S5 Fig**). Here, external stimulation of the FS population (a rightward translation of the FS nullcline) decreases steady-state firing rates of both the PC and FS populations.

In contrast, to reproduce firing rates corresponding to increased FS rheobase, the gain of the PC population increased such that *B*_*E*_*W*_*EE*_ exceeds a value of 1 (**Fig 5**). When stimulating the PC population of the FS model to approximate conditions of increased FS rheobase, the model underwent a Hopf bifurcation and exhibited sustained oscillations (**Fig 5**). Furthermore, both the FS-PC phase portrait and the model’s response to external inhibitory stimulation demonstrated features consistent with an ISN (**Figs 5D** and **S5**). Compared to the NFS model, the PC nullcline has a rightward inflexion (**Fig 5**). With sufficient external input, the FS nullcline passes above this inflexion, and stimulation of the FS population leads to a reduction in steady-state firing rates of both PC and FS populations. Therefore, to reproduce firing rates of the spiking network, the FS and NFS rate models exert different influence over the gain of the PC population. Notably, reduced FS excitability is associated with increased PC gain that generates characteristics of an inhibition-stabilised state.

### Excitatory gain modulation in the detailed network

The findings from our rate models and the existence of a paradoxical response to inhibitory perturbation in the spiking network provide indirect evidence that reduced FS excitability enhances the effective synaptic weight (*B*_*E*_*W*_*EE*_) of the PC population. We sought to directly confirm this finding within our spiking network, and to then investigate mechanisms through which *B*_*E*_*W*_*EE*_ is modulated via the FS interneuron population.

**Fig 6:**
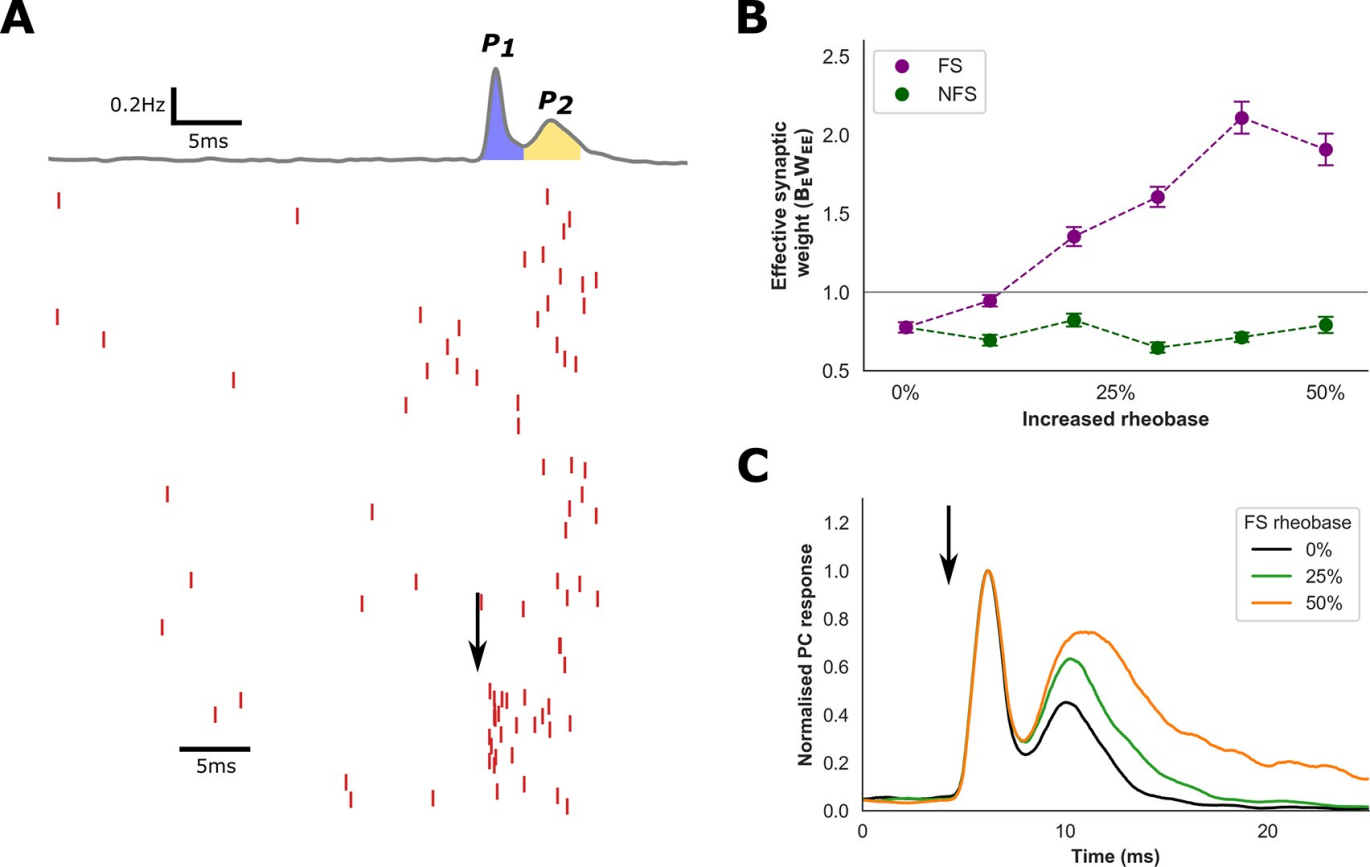
PC gain modulation in the spiking network. **A)** Raster plot of PC spiking (spikes denoted in red) and average population response (top trace, grey) in response to a brief stimulus applied to 20% of the PC population (black arrow). The stimulus elicited a bi-modal population response: the first peak (P_1_) in response to the stimulus and the second peak (P_2_) from PC-to-PC interactions. The effective synaptic weight was estimated by calculating the ratio of the area under the curves of P_2_ (yellow) to P_1_ (blue). **B)** Estimated effective synaptic weight (B_E_W_EE_) under conditions of increased FS and NFS rheobase. FS rheobase increased B_E_W_EE_ above 1, consistent with excitatory subnetwork instability, and produced a significant increase in B_E_W_EE_ compared to NFS rheobase (P < 0.001). **C)** Examples of normalised average population response to the perturbation (arrow) following increased FS rheobase.

To measure the effective synaptic weight in our detailed network, we perturbed a fraction (20%) of the PC population with a brief current stimulus and calculated the average response of the network (**Fig 6**). An estimate for *B*_*E*_*W*_*EE*_ was obtained by measuring the ratio of PC neurons recruited via PC-to-PC interactions compared to spikes directly evoked by the stimulus. According to our hypothesis, if *B*_*E*_*W*_*EE*_ is less than 1, fewer PC neurons should be recruited through PC-to-PC interactions. Conversely, if *B*_*E*_*W*_*EE*_ is greater than 1, more PC neurons should be recruited through PC-to-PC interactions, suggestive of excitatory subnetwork instability.

At baseline, fewer PC neurons were recruited through PC-to-PC interactions, consistent with an estimated value of *B*_*E*_*W*_*EE*_ under 1 (**Fig 6**). As FS rheobase is increased, we observed a progressive increase in *B*_*E*_*W*_*EE*_ that exceeded 1, with more PC neurons recruited through PC-to-PC connections (*P* < 0.001 for greater than 10% increase in rheobase, one-way ANOVA with post-hoc Tukey’s test). Furthermore, *B*_*E*_*W*_*EE*_ was significantly higher for increased FS compared to NFS rheobase (*P* < 0.001, Welch’s *t*-test, for rheobase values of 10, 20, 30, 40 & 50%). In contrast, increased NFS rheobase did not increase *B*_*E*_*W*_*EE*_ above 1, suggesting that the excitatory subnetwork remains stable under these conditions. These findings are consistent with results from our simple model and, given that synaptic weights in the model are fixed, provide further evidence that reductions of FS excitability enhance PC gain.

### Mechanisms of gain modulation

We next investigated potential mechanisms through which the excitability of the FS population can modulate PC gain. Since we can assume the intrinsic and synaptic properties of the PC model are fixed, we surmise that changes of gain are related to input conditions to which the PC population is exposed. Therefore, we explored the influence of three changes to input conditions associated with increased FS rheobase: reductions of inhibitory current, delayed feedback inhibition, and stronger excitatory input correlations.

First, we note that increased FS rheobase initially reduces total mean inhibitory current onto the PC population (**S6 Fig**, also appreciated by an increase of EI ratio in **Fig 3**). A change to the balance of excitatory and inhibitory inputs have previously been demonstrated to enhance neuronal gain, and it is possible reductions of inhibition may exert a similar influence in our network[63,67,68]. Therefore, to test if a reduction of inhibitory input can enhance gain in the absence of input correlations, a network containing only PC neurons (the “PC network”) was created and stimulated with Poisson-distributed inputs that reproduce the conditions of the three-population network (**S6 Fig**). PC gain was assessed by stimulating a fraction of the PC neurons and recording the average spiking response of the network (**S6 Fig**). Here, we used a simplified measure of gain: since the PC network contains no inhibitory feedback, a value of *B*_*E*_*W*_*EE*_ above 1 will generate persistent excitatory activity, which we observed after a ~10% reduction of inhibition (**S6 Fig**). However, although reductions of inhibitory input caused by increased FS rheobase may contribute to enhanced PC gain, increased NFS rheobase in fact produced greater reductions of inhibitory input yet did not modulate PC gain (**S6 Fig**). Therefore, there must also exist other mechanisms associated with increased FS rheobase that enhance PC gain.

**Fig 7:**
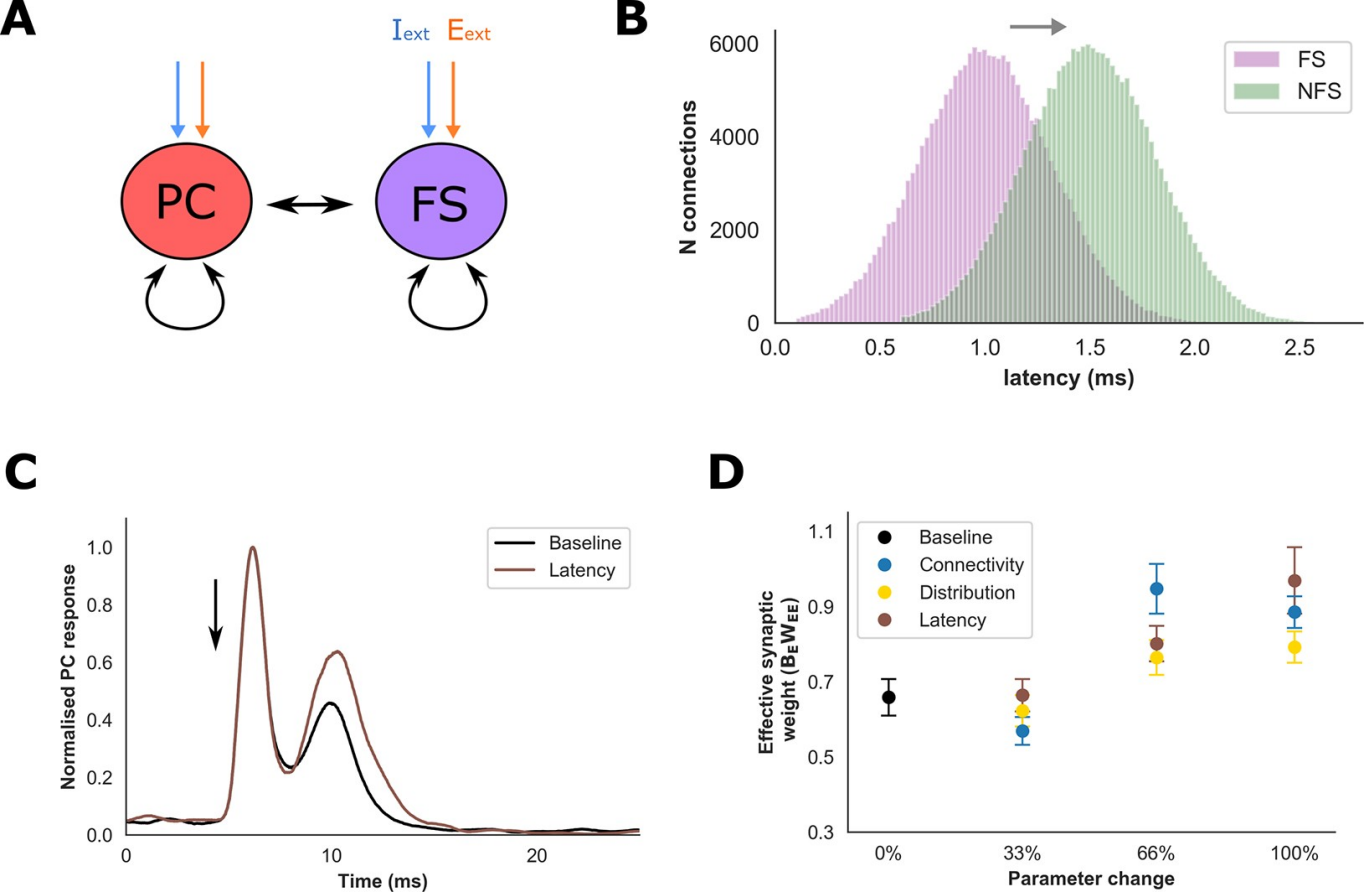
Interneuron properties and PC gain modulation. **A)** A network with a homogeneous FS interneuron population was developed to explore the role of interneuron synaptic properties upon PC gain. **B)** Histogram of FS-to-PC synaptic latencies before (purple, ‘FS’) and after (green, ‘NFS’) modification to resemble NFS latencies. Connectivity and synaptic distribution were modified in a similar manner. **C)** Example of normalised average population response to the perturbation (arrow) following modification of interneuron latency. **C)** Estimated effective PC synaptic weight (B_E_W_EE_) within the FS network after modifying interneuron properties to resemble the NFS population (100% represents NFS values). An increase in estimated B_E_W_EE_ was observed across all parameters, and at 100% latency and connectivity generated a significant change compared to baseline (P < 0.01, Welch’s t test).

Mean synaptic latencies between PC and FS interneurons are shorter compared to NFS interneurons in our detailed network (1.0 ms and 1.5 ms, respectively), and it is possible that delayed feedback inhibition may contribute to differential PC gain modulation. To test this hypothesis, a network with a homogeneous interneuron population comprising just FS neurons was developed (the “PC-FS network”). PC and FS parameters were otherwise identical to the original network (**Fig 7**). To isolate the role of feedback inhibition upon PC gain, we estimated the value of *B*_*E*_*W*_*EE*_ after progressively increasing mean synaptic latency between the PC and FS population to 1.5 ms (denoted 100% of the NFS value, **Fig 7B** and **7D**) whilst preserving all other synaptic properties. Prolongation of synaptic latency produced a progressive increase in mean estimated *B*_*E*_*W*_*EE*_, and *B*_*E*_*W*_*EE*_ was significantly greater than baseline values (*P* < 0.01, Welch’s t-test, **Fig 7**). Therefore, an isolated prolongation of feedback inhibition, similar to that provided by the NFS population, can indeed enhance PC gain relative to the faster feedback inhibition provided by the FS population. We also used the PC-FS network to explore the contribution of differences in synaptic connectivity and distribution between the FS and NFS populations upon PC gain modulation. Both connectivity and distribution also increased *B*_*E*_*W*_*EE*_, but only connectivity generated a significant change compared to the baseline value (**Fig 7D**.

**Fig 8:**
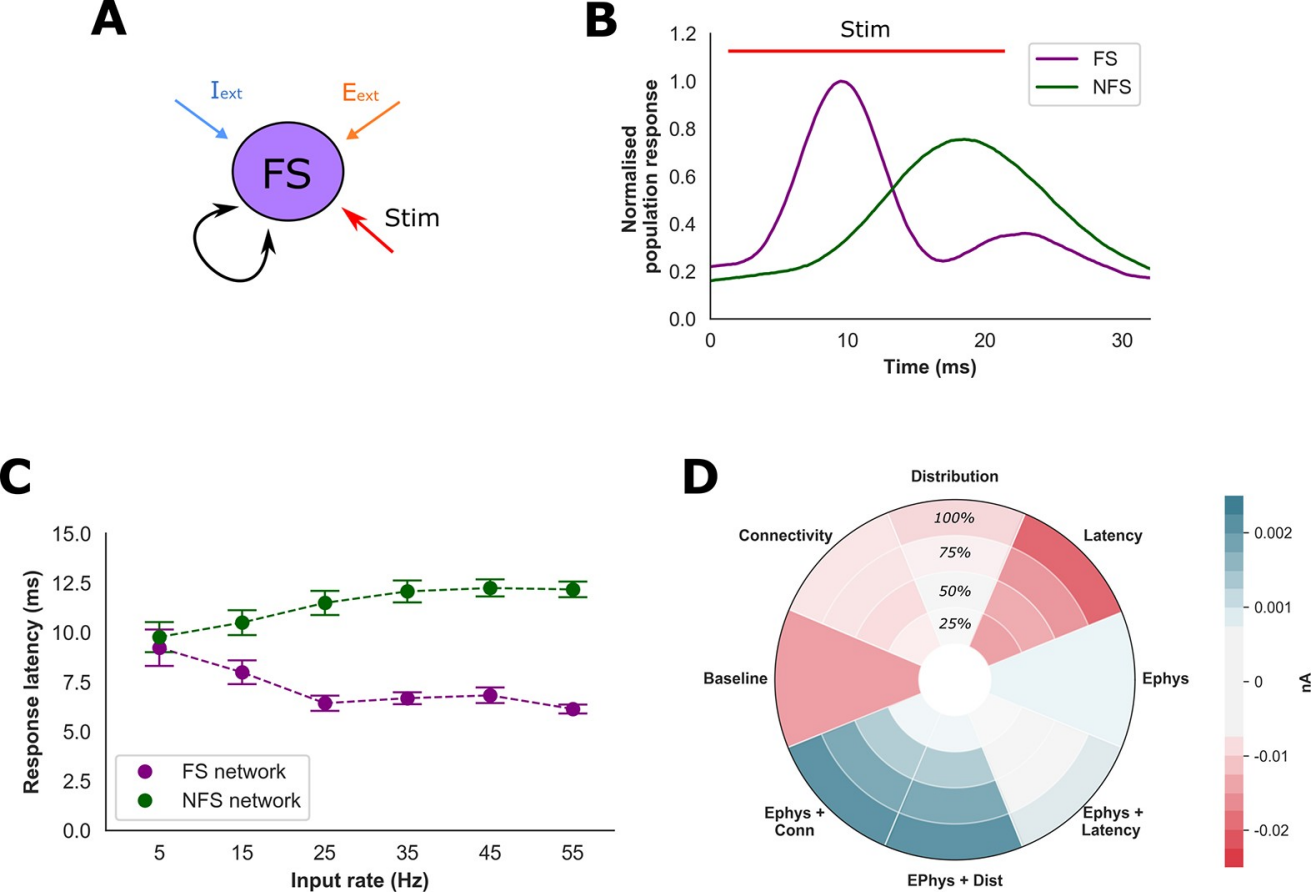
Intrinsic interneuron electrophysiological properties. **A)** To explore the influence of interneuron electrophysiological properties, a network of just FS or NFS interneurons was developed and stimulated with excitatory synaptic input of increasing mean frequency (‘Stim’). **B)** Normalised average interneuron population responses to synaptic input demonstrating differences in mean response latency between the FS and NFS populations. **C)** Mean latency to peak response as a function of excitatory input frequency. Excitatory inputs generated a more rapid-onset response within the FS compared to NFS population (P < 0.001 for input rates above 15 Hz). **D)** Efficacy of modifying intrinsic electrophysiological and synaptic NFS interneuron properties upon rescuing the original three-population network from an ISN regime. The spiking network was first initialised into an ISN by increasing FS rheobase by 50% (‘Baseline’). Specific properties of the NFS population were then modified to resemble the FS population, and a paradoxical response assessed by perturbing the FS and NFS populations, calculating the change of inhibitory current onto the PC population and then averaging these responses (paradoxical response denoted by negative values in red, e.g., **Fig 4**). Synaptic connectivity, distribution and latency were adjusted incrementally to resemble the FS population (25, 50, 75 & 100%).

Considering these findings, we wondered if the intrinsic electrophysiological properties of FS and NFS interneurons are also relevant for feedback inhibition and gain modulation. To address this question, we created networks containing only FS or NFS interneurons (the “FS interneuron network” and “NFS interneuron network”, **Fig 8**). The FS and NFS interneuron networks were then stimulated with excitatory synaptic input designed to replicate fluctuations of input from the PC population (**Fig 8A** and **8B**). Although both the FS and NFS interneuron networks generated similar total spikes to excitatory inputs (**S6 Fig**) we observed a significantly shorter response latency in the FS interneuron network of over 2 ms (*P* < 0.001 for input rates above 0.5 Hz, Welch’s *t* test, **Fig 8**). Importantly, this value is greater than the difference in mean synaptic latency between the FS and NFS populations that was sufficient to enhance PC gain, implying that rapid intrinsic response properties to synaptic input also afford the FS population influence over PC gain (**Fig 7B** and **7C**).

Finally, we explored if the emergence of excitatory spike correlations can also enhance gain. This was achieved by stimulating PC neurons with artificial spike trains containing progressively stronger correlations that approximated those arising within our network during increased FS rheobase (**S7 Fig**). We found that spike correlations significantly enhanced PC excitability: at an input rate that elicits a mean frequency of 10 Hz for uncorrelated inputs, a spike cross-correlation of 0.0025 elicited a mean frequency of 29.5 Hz (*P* < 0.001, Welch’s *t* test, **S7 Fig**). Furthermore, these findings held regardless of the distribution of spikes across different regions of the PC model, consistent with previous studies demonstrating gain modulation through enhanced fluctuations of membrane potential [69,70]. Indeed, we found that reductions of FS excitability produced a large and significant increase in excitatory current variance onto the PC population (*P* < 0.001 for FS rheobase ≥ 10%, Welch’s *t* test, **S7C** and **S7D Fig**).

In summary, three mechanisms together enhance gain within the PC population of our network: reduced inhibitory input, delays of feedback inhibition, and excitatory input correlations. An important difference between the FS and NFS population is the rate at which they can provide feedback inhibition in response to excitatory input, a property associated with both synaptic delay and their intrinsic electrophysiological properties. Suppressing FS excitability will preferentially shift feedback inhibition within the network onto the NFS population. Since NFS-mediated inhibition is provided at longer latencies, this in turn may enhance gain within the PC population to promote a transition in network state.

## Discussion

Experimental recordings of cortical activity have revealed a diversity of network states that are thought to enrich the brain’s computational capabilities and allow for context-dependent cognitive flexibility[1,71–74]. Neuronal and synaptic heterogeneity provide a substrate from which diverse regimes of activity can emerge and interneuron subtypes, in particular, appear to play an important role for promoting different network states[1,2,42,75]. To explore the impact of interneuron excitability upon cortical activity, we used a spiking network model that incorporated synaptic and electrophysiologic features of FS and NFS interneuron subtypes. We found that reductions of FS excitability promoted network activity characterised by enhanced spike correlations and population synchrony, and features of an inhibition stabilised state associated with increased gain within the PC population.

Our findings suggest that FS interneurons can preferentially modulate gain within the excitatory population to enable transitions in network state. We found indirect evidence for enhanced PC gain through a paradoxical response to inhibitory stimulation, a shift to lower EI ratios, and a Wilson-Cowan model that reproduced firing rates of the detailed network through modulation of excitatory gain. These findings were confirmed by direct perturbation of the PC population, and further analysis revealed several mechanisms through which reduced FS excitability can modulate gain (**Figs 6 and 8**). Interestingly, although reductions of total inhibitory input may contribute, this alone was insufficient to account for enhanced PC gain as reduced NFS excitability produced greater reductions of inhibitory input (see **S6 Fig**)[68]. Indeed, we found that gain was also modulated through temporal variability of feedback inhibition and the emergence of spike correlations–mechanisms which have been demonstrated previously to modify the excitability of pyramidal cell populations and contribute to orientation tuning in visual cortex[63,76,77]. Although membrane potential fluctuations associated with correlated excitatory or inhibitory inputs can enhance gain, stronger temporal coupling between excitatory and inhibitory inputs can dampen excitability[69,70]. Suppression of FS excitability in our network transfers the dominant mode of inhibition onto the NFS population which is characterised by more prolonged feedback inhibition. Therefore, a transfer to NFS-mediated inhibition lengthens the temporal window for rapid PC-to-PC synaptic interactions to emerge.

By using a network with a homogeneous interneuron population and then introducing features specific for FS and NFS interneurons, we found that differences in synaptic latency, connectivity, and the intrinsic electrophysiological properties of the interneuron models were of relevance for PC gain modulation (**Figs 7D** and **8**). A lingering question is the impact of these interneuron properties upon a paradoxical response to stimulation of the inhibitory population. In addition to enhanced PC gain, ISNs also require the presence of other complex network features, such as strong coupling between PC and interneuron populations[15,66]. Therefore, we tested the influence of interneuron properties upon the ability to ‘rescue’ the spiking network from an inhibition stabilised state (**Fig 8**). Here, we found that differences in the intrinsic electrophysiological properties of the interneuron models appeared to exert greatest influence. Interestingly, although increasing synaptic latency enhanced PC gain, we also observed that shortening synaptic latency could promote a stronger paradoxical response (**Fig 8**). This counterintuitive finding may be explained by the fact that a shortened latency strengthens interactions between the PC and interneuron populations and, provided PC gain is sufficiently high, enhance a paradoxical response to inhibitory stimulation. Overall, our findings confirm that specific biophysical properties of FS interneurons afford them a powerful inhibitory influence over network activity[3,28,63]. Furthermore, our model suggests that the ability of FS interneurons to provide rapid feedback inhibition is of particular importance in this respect.

A reduction of FS excitability also produced a state transition within a network containing simplified Izhikevich PC models, supporting the generalisability of our results. Although PC gain in the Izhikevich network could be modulated through similar mechanisms as the detailed network, the Izhikevich network exhibited weaker pair-wise spike correlations and population oscillations, and the timing of excitatory and inhibitory inputs had less impact upon neuronal excitability (**S8 Fig**). In light of these variations in network behaviour, we also investigated differences in the biophysical properties of the detailed and Izhikevich PC model and found that a crucial distinction is the presence of a shorter effective membrane time constant in the detailed PC model that confers sensitivity to input correlations (**S9 Fig**)[78]. Furthermore, it has been shown that pyramidal neurons that are sensitive to correlated (coincident) inputs can exhibit more precise spike-time synchronisation[79]. This may account for the emergence of stronger synchrony and spike correlations in the network containing the detailed PC model (**Fig 3**). Therefore, although FS interneurons could mediate a state transition in both the detailed and Izhikevich networks, the spike features and mechanisms that drive this transition are to some extent dependent upon the intrinsic properties of the PC population[79,80]. In the detailed PC model, a shorter time constant allows variation of input timing to modulate gain, and promoted correlated spiking as the network undergoes transition.

This study provides further evidence for the existence of inhibition stabilised states in networks with biologically realistic connectivity, synaptic parameters and cellular composition[16,66]. We also tested the generalisability of these findings under different conditions by stimulating the model with just excitatory inputs (**S4 Fig**). Our network also exhibits several features consistent with experimental cortical recordings, including irregular and asynchronous firing, inhibition-dominated synaptic input conductances, and fast temporal correlations between excitatory and inhibitory inputs[10,49,59,61]. One difference is the proportion of excitatory input derived from recurrent network connections. In our model, only ~3% of excitatory current is obtained from within the network, whereas it has been estimated experimentally that over 50% of excitatory inputs are derived from recurrent cortical connections (**Fig 2**)[81]. Furthermore, in theoretic models of ‘balanced’ networks, external input is assumed to be of similar order of magnitude to recurrent input[58]. These differences may relate to the size of our network and the use of biophysically realistic synaptic weights that were not tuned to recreate certain input conditions. We used cell numbers that approximated a cortical column layer, whereas experimental estimates of recurrent input have been derived after suppressing activity throughout large regions of cortex[81]. Therefore, our model’s external stimulation would implicitly contain ‘recurrent’ cortical inputs under this scenario, and it is possible a more expansive network may closer approximate balanced conditions without a need to scale synaptic weights.

Our results suggest that FS interneurons are uniquely positioned to enable transitions in cortical state characterised by large changes in gain within the excitatory population. Strong coupling between excitatory neurons endows cortical networks with certain computational abilities including nonlinear response summation to external input, multi-stable dynamics and persistent activity associated with short-term memory formation[15,58,82]. Consequently, neurotransmitters that selectively inhibit FS interneurons may promote this regime for the execution of cognitive tasks[23]. High excitatory gain is also associated with the emergence of epileptic seizures, and therapeutic strategies augmenting FS activity have been demonstrated to be an effective method for seizure termination[83–85]. Although FS interneurons in our network could also mediate the transition between a non-ISN and ISN, it remains unclear if cortical networks operate as a non-ISN regime, with recent studies demonstrating the existence of ISNs across multiple cortical regions and behavioural conditions[15,16]. Interestingly, it has been shown that the extent of the paradoxical response to perturbation of the inhibitory population, measured by the magnitude of change of inhibitory firing rate, may be dependent upon brain state such as the degree of arousal[16]. Therefore, it is possible that under states of low arousal, such as anaesthesia or deep sleep, regions of cortex may indeed operate as a non-ISN. If so, and considering our results, FS interneurons could play a central role in the transition between states of arousal.

This study contains several limitations that may be addressed in future work. Since we incorporated broad biological differences in FS and NFS interneurons our conclusions may not generalise to specific brain regions as cell composition and connectivity vary widely across cortical regions and layers[3,86]. Furthermore, although we utilised spiking models with electrophysiological differences between neuron subtype our models did not possess complex dendritic morphology, limiting the realism of synaptic input integration[87]. In this study we have explored the role of two interneuron populations that approximate the features found in PV and SST subtypes. Notably, we have not investigated vasoactive intestinal peptide-expressing (VIP) interneurons that have been shown to mediate a disinhibitory circuit[3]. Activity of the VIP population, therefore, may further modify the influence of FS and NFS interneuron subtypes upon networks dynamics.

## Supporting information

S1 Fig **A)** Breakdown of excitatory and inhibitory input conductances onto the PC population. Net conductance (E+I) was received from inhibitory input sources (inhibition-dominated). **B)** Excitation and inhibitory synaptic input currents onto the FS (left) and NFS (right) population during baseline conditions. **C)** Mean firing rates of each population with increased NFS rheobase. As NFS firing rates approach 0 there is a compensatory increase in FS firing rates (raster plot on right corresponds to NFS rheobase of 100%). **D)** Cross correlation of excitatory and inhibitory synaptic input conductance’s onto the PC population with increased NFS (left) and FS (right) rheobase (corresponding to PC-I in **Fig 1**).
(TIF)

S2 Fig Change in total inhibitory current onto the PC population with external stimulation of the FS and NFS populations during baseline conditions (**A**) and with increased NFS rheobase (**B-C**). A reduction of inhibitory current in the presence of external FS/NFS stimulation was not observed, consistent with a non-ISN regime.
(TIF)

S3 Fig Characteristics of the Izhikevich network in response to modulation of the FS and NFS populations. Increased FS rheobase evoked higher PC firing rates (**A**) compared to increased NFS rheobase. Increased FS rheobase also generated stronger population oscillations (**B**) compared to baseline (P < 0.001 for FS rheobase above 10%, one-way ANOVA with post hoc Tukey test) and a significant increase in spike correlations compared to baseline above 30%, although the magnitude of spike correlations was much smaller compared to the original model (mean of 0.00048 vs 0.012, respectively, for increased FS rheobase of 50% in the Izhikevich vs original network. Increased FS rheobase was also associated with a reduction of EI balance (**D**) and a more pronounced paradoxical response to inhibitory stimulation (**E**) consistent with an ISN.
(TIF)

S4 Fig Characteristics of the network stimulated by just excitatory synaptic input in response to modulation of the FS and NFS populations. Increased FS rheobase evoked higher PC firing rates (**A**) compared to increased NFS rheobase. Increased FS rheobase also generated stronger population oscillations (**B**) and pairwise spike correlations (**C**) compared to baseline (P < 0.001 for FS rheobase above 5%, one-way ANOVA with post hoc Tukey test). A significant increase in either population oscillations or spike correlations compared to baseline was not observed with increased NFS rheobase. Again, increased FS rheobase was associated with a reduction of EI balance (**D**) and a more pronounced paradoxical response to inhibitory stimulation (**E**).
(TIF)

S5 Fig **A)** Progression of fitness values with successive iterations during optimisation of the rate model. **B)** PC gain (B_E_) was recalculated in terms of net current for increasing FS and NFS rheobase values (purple & green respectively and corresponding rheobase values labelled). The change in PC gain (B_E_) with net input current was fitted with a sigmoidal function (**B**, right). Since net input current during conditions of increased FS rheobase shifted to more hyperpolarised values as the network transitioned into an ISN regime (evident by the leftward deflection of the PC gain curve) only the initial upstroke in PC gain was used to ensure that B_E_ is a one-to-one function of net input current for the purposes of the rate model. **C)** Change in inhibitory current onto the PC population of the rate model, during external stimulation of the FS/NFS population. Conditions at baseline and for the NFS/FS rate models correspond to **Fig 4**. A paradoxical reduction of inhibitory current onto the PC population, despite external stimulation of the inhibitory population, was observed in the FS model but not during baseline conditions or the NFS model, confirming the presence of an ISN regime.
(TIF)

S6 Fig **A)** Change in total inhibitory current onto the PC population with increased FS rheobase. A reduction of inhibitory current is observed with increased FS rheobase before the network transitions to an ISN. Although increased NFS rheobase was associated with greater reductions of inhibitory current these changes did not produce an increase in PC gain (**Fig 6**). **B)** To investigate the impact of reductions of inhibitory input upon PC gain, a network of PC neurons (“the PC network”) was developed with external stimulation that reproduced the mean inhibitory and excitatory current of the original three-population network. **C)** Mean population response of the PC network in response to a brief stimulus (black arrow) after a reduction of external inhibition (coloured traces). A reduction greater than ~10% was sufficient to generate unrestrained excitatory activity, demonstrated by the raster plot for one simulation in **D**. **E)** Total number of spikes generated by the FS and NFS interneuron networks in response to excitatory stimuli shown in **Fig 8**. We observed small differences in total spikes elicited by each interneuron population, and at lower input rate fluctuations more spikes were elicited by the NFS interneuron network.
(TIF)

S7 Fig **A)** Raster plots for spike trains generated using a Poisson-process (baseline) and after introducing pair-wise spike correlations. Both spike trains have identical mean firing rate. **B)** Input-output relationship of PC neurons in response to generated spike trains of increasing rate (x-axis) and pair-wise spike correlation (colours). Inputs were applied to either the soma, dendrite or distributed equally across both compartments (“Both”). The presence of stronger spike correlations enhanced the excitability of the PC neuron model: at an input rate that elicits a spike frequency of 10Hz for uncorrelated inputs, a spike cross-correlation of 0.0025 elicited a frequency of 29.5Hz (P < 0.001, Welch’s t-test). Error bars denote s.e.m. **C)** Variance of excitatory synaptic input derived from internally generated excitatory network activity during conditions of increased FS and NFS rheobase. **C)** Greater variance with increased FS rheobase is appreciated visually from the traces of mean-normalised excitatory current input during baseline (left) and increased FS/NFS rheobase (right, purple/green traces respectively).
(TIF)

S8 Fig Mechanisms of gain modulation in the Izhikevich network. **A)** Input-output relationship of the single compartment Izhikevich PC model in response inputs of increasing rate and spike correlation (colour coded). Compared to the original PC model (**S7 Fig**), correlated synaptic inputs had less impact upon the excitability of the Izhikevich PC model: at a rate that elicits a spike frequency of 10Hz for uncorrelated inputs, a spike cross-correlation of 0.0025 elicited a frequency of only 13Hz (P < 0.001, Welch’s t test). **B)** Mean population response of the Izhikevich PC network in response to a brief stimulus (arrow) after a reduction of external inhibition. Similar to the PC network in **S6 Fig**, reduced inhibition generated unrestrained excitatory activity consistent with enhanced PC-to-PC synaptic gain. **C**) To explore the timing of inhibition, both the detailed and Izhikevich (simple) PC models were stimulated with identical excitatory and inhibitory spike trains, and a delay introduced to the inhibitory inputs of increasing duration. Here, inhibitory spikes have a mean delay of 2.0 ms compared to excitatory spikes. Input-firing rate relationship of the detailed (**B**) and Izhikevich (**C**) PC models with lengthening of inhibitory delays (color-coded). We found that the detailed model was sensitive to small delays of inhibitory input under 1ms: a delay of 0.5 ms increased the mean spike rate from 5 Hz to 34 Hz (P < 0.001, Welch’s t-test). Furthermore, delays greater than 0.5 ms had insignificant further impact on excitability. Although the Izhikevich PC model was also sensitive to inhibitory delays, the magnitude of the change in excitability was considerably smaller: a delay of 10 ms increased the mean spike rate from 5 to 11 Hz (P < 0.001). Changes to excitability occurred across a wider range of values, and delays greater than 10 ms did not exert a significant additional impact on excitability.
(TIF)

S9 Fig **A)** Time-voltage trace of the original (top) and Izhikevich (bottom) PC model in response to excitatory synaptic inputs (arrows). Membrane voltage decays more rapidly in the original PC model. **B)** Mean input-firing rate relationship of the Izhikevich model in response uncorrelated (solid line) and correlated (pair-wise cross-correlation of 0.005, dashed line) input after reducing membrane capacitance to 50 and 25% (blue and orange traces, respectively). Reducing the membrane time constant of the Izhikevich model by reducing membrane capacitance increases the sensitivity of the model to correlated input. For instance, the mean spike threshold at 25% capacitance decreased from 19.6 to 3.3Hz for inputs with a pairwise cross-correlation of 0.005, compared to 4.9 to 3.3Hz at 100% capacitance. **C)**The impact of other mechanisms for conferring sensitivity to correlated inputs in the detailed PC model was also explored by reducing the conductance of each ion channel mechanism, and then re-calculating the input-output relationship in response to uncorrelated (solid line) and correlated (dashed line) input. No mechanisms could abolish sensitivity to input correlations, further supporting the importance of the rate of decay of synaptic input. Here, enhanced sensitivity to correlated inputs was clearly retained despite complete removal of the CaH and Ih channels. Reductions of KP and NaT conductance below 75% and 50% respectively produced a loss of sustained firing.
(TIF)

S1 Table Stimuli, features and objective values used to constrain the Pyramidal Cell model. AP: action potential; BAP: back-propagating action potential; AHP: after-hyperpolarization potential; ISI: inter-spike interval; ms: millisecond; mV: millivolt.
(DOCX)
