# Supplementary figures and images for: State transitions through inhibitory interneurons in a cortical network model
Source: PLoS Comput Biol. 2021 Oct 15;17(10):e1009521. doi: 10.1371/journal.pcbi.1009521 (PMC8550371; doi:10.1371/journal.pcbi.1009521)

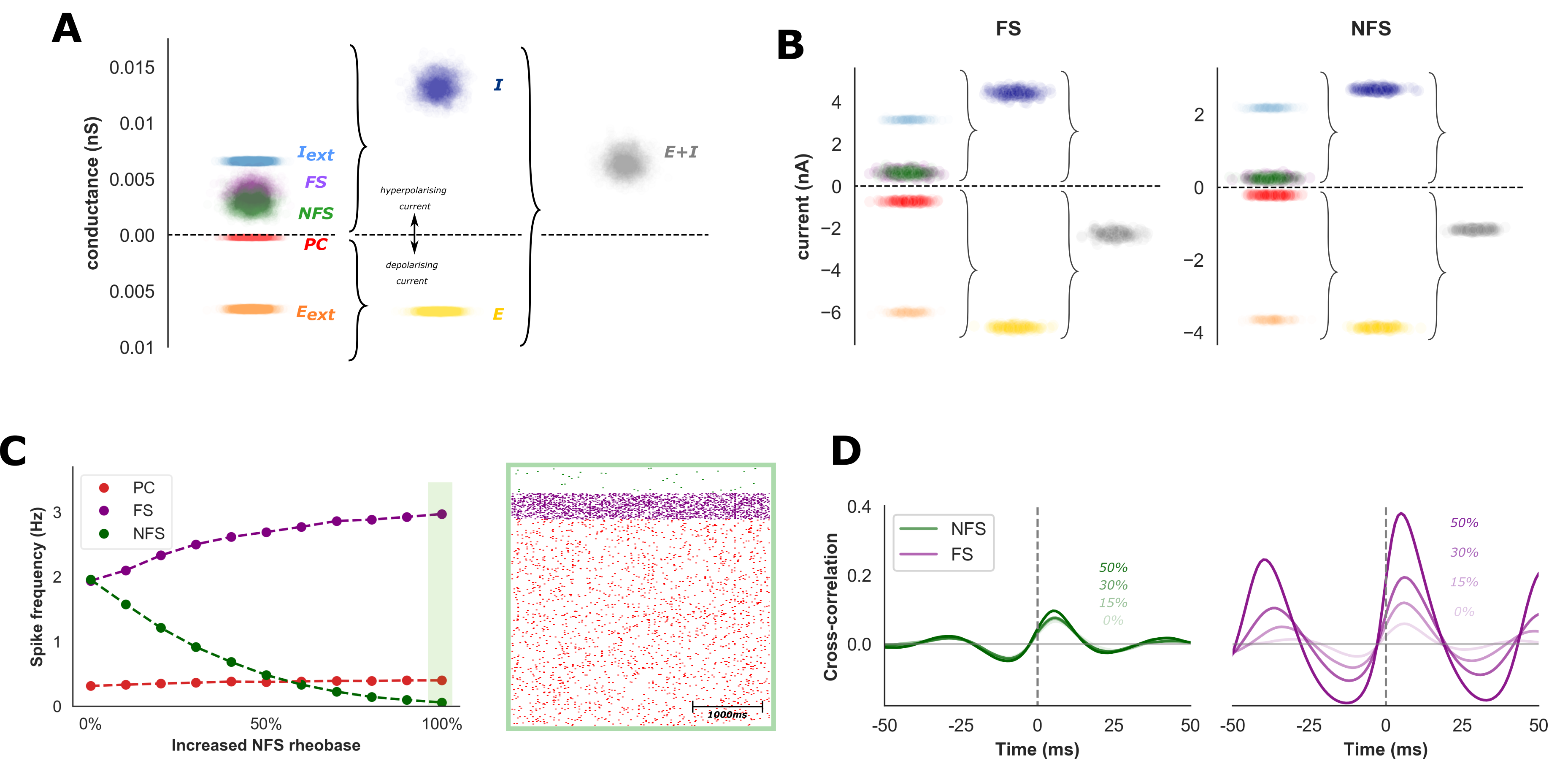

Supplement: S1 Fig — A) Breakdown of excitatory and inhibitory input conductances onto the PC population. Net conductance (E+I) was received from inhibitory input sources (inhibition-dominated). B) Excitation and inhibitory synaptic input currents onto the FS (left) and NFS (right) population during baseline conditions. C) Mean firing rates of each population with increased NFS rheobase. As NFS firing rates approach 0 there is a compensatory increase in FS firing rates (raster plot on right corresponds to NFS rheobase of 100%). D) Cross correlation of excitatory and inhibitory synaptic input conductance’s onto the PC population with increased NFS (left) and FS (right) rheobase (corresponding to PC-I in Fig 1). (TIF) [file pcbi.1009521.s001.tif]

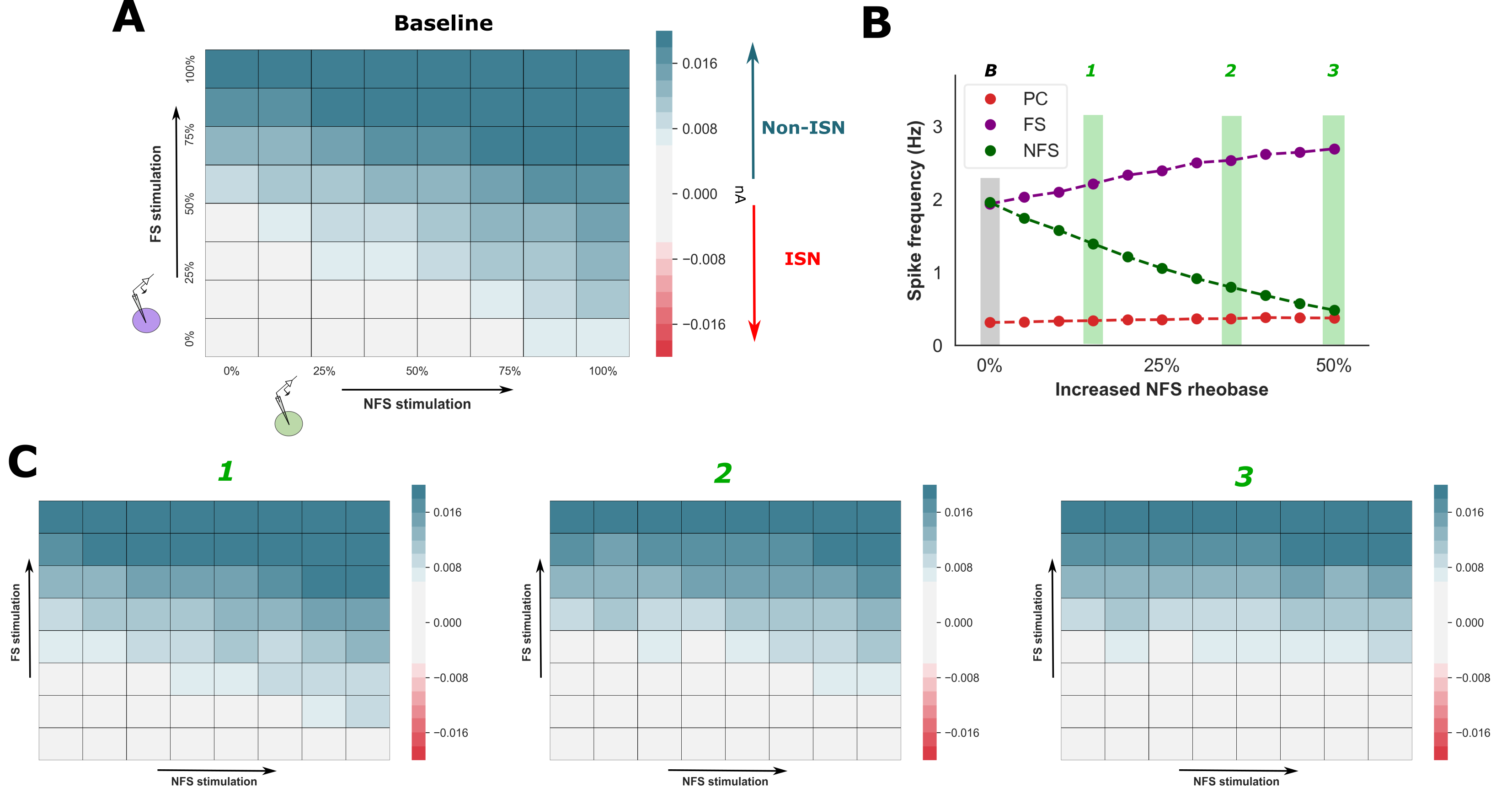

Supplement: S2 Fig — Change in total inhibitory current onto the PC population with external stimulation of the FS and NFS populations during baseline conditions (A) and with increased NFS rheobase (B-C). A reduction of inhibitory current in the presence of external FS/NFS stimulation was not observed, consistent with a non-ISN regime. (TIF) [file pcbi.1009521.s002.tif]

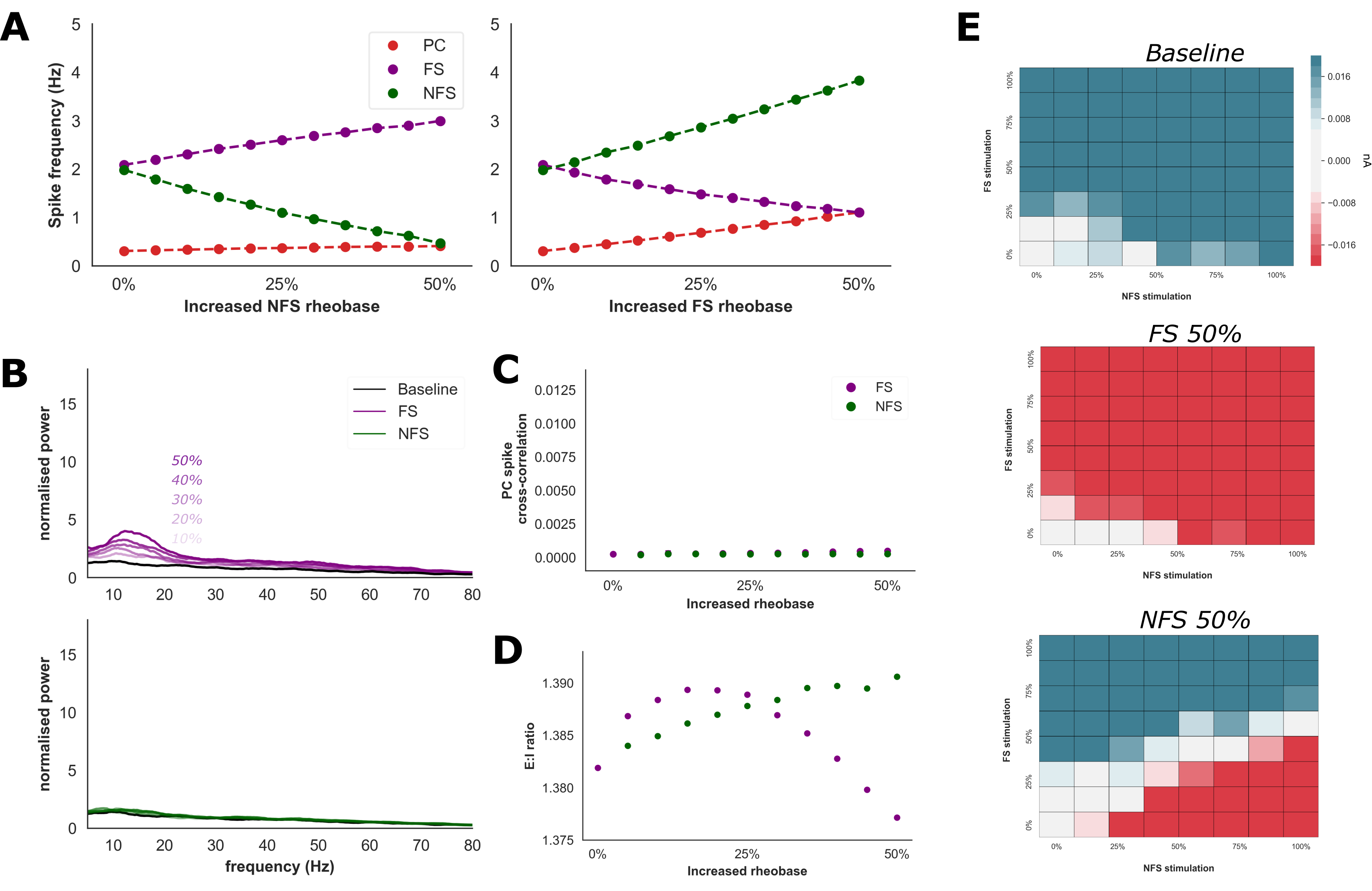

Supplement: S3 Fig — Increased FS rheobase evoked higher PC firing rates (A) compared to increased NFS rheobase. Increased FS rheobase also generated stronger population oscillations (B) compared to baseline (P < 0.001 for FS rheobase above 10%, one-way ANOVA with post hoc Tukey test) and a significant increase in spike correlations compared to baseline above 30%, although the magnitude of spike correlations was much smaller compared to the original model (mean of 0.00048 vs 0.012, respectively, for increased FS rheobase of 50% in the Izhikevich vs original network. Increased FS rheobase was also associated with a reduction of EI balance (D) and a more pronounced paradoxical response to inhibitory stimulation (E) consistent with an ISN. (TIF) [file pcbi.1009521.s003.tif]

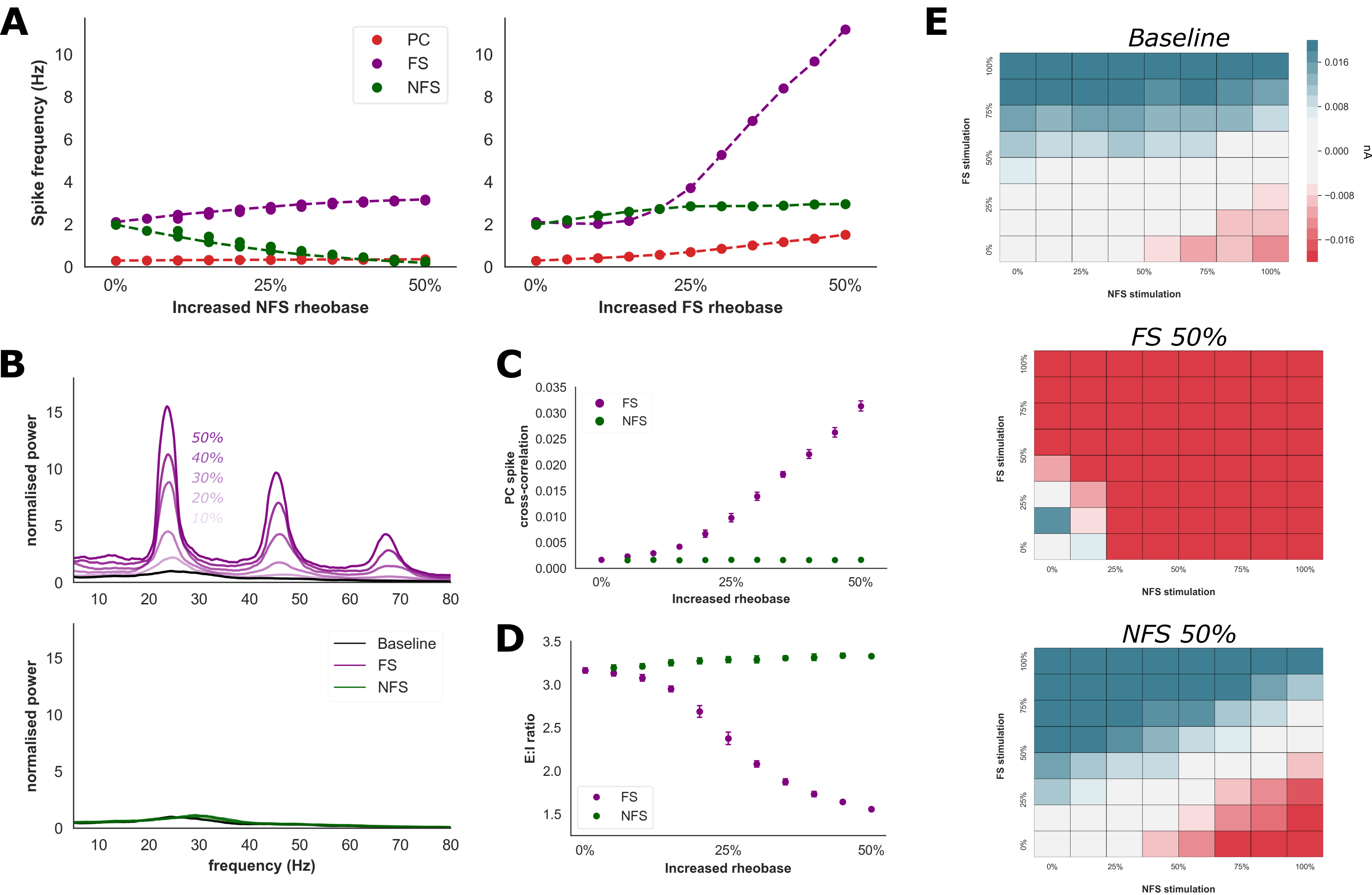

Supplement: S4 Fig — Increased FS rheobase evoked higher PC firing rates (A) compared to increased NFS rheobase. Increased FS rheobase also generated stronger population oscillations (B) and pairwise spike correlations (C) compared to baseline (P < 0.001 for FS rheobase above 5%, one-way ANOVA with post hoc Tukey test). A significant increase in either population oscillations or spike correlations compared to baseline was not observed with increased NFS rheobase. Again, increased FS rheobase was associated with a reduction of EI balance (D) and a more pronounced paradoxical response to inhibitory stimulation (E). (TIF) [file pcbi.1009521.s004.tif]

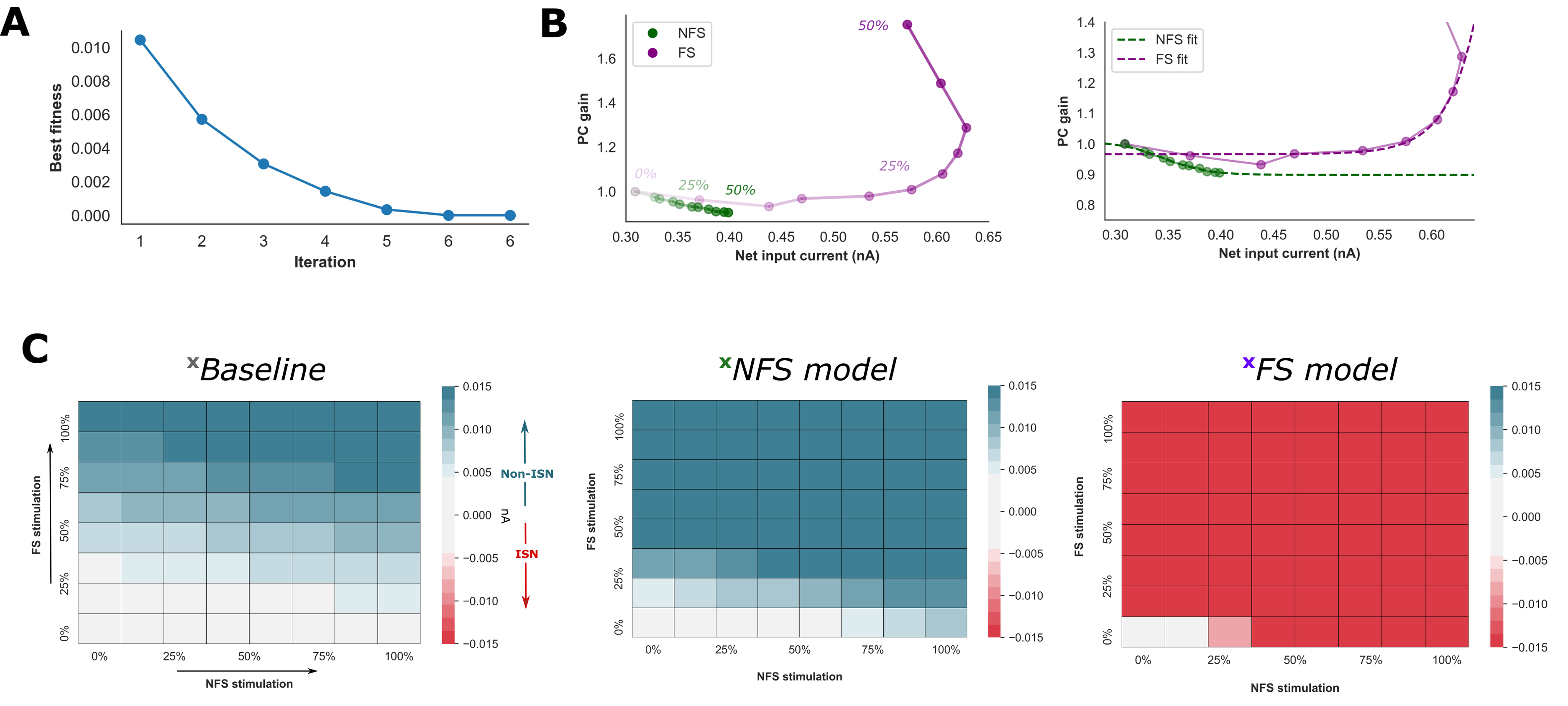

Supplement: S5 Fig — A) Progression of fitness values with successive iterations during optimisation of the rate model. B) PC gain (BE) was recalculated in terms of net current for increasing FS and NFS rheobase values (purple & green respectively and corresponding rheobase values labelled). The change in PC gain (BE) with net input current was fitted with a sigmoidal function (B, right). Since net input current during conditions of increased FS rheobase shifted to more hyperpolarised values as the network transitioned into an ISN regime (evident by the leftward deflection of the PC gain curve) only the initial upstroke in PC gain was used to ensure that BE is a one-to-one function of net input current for the purposes of the rate model. C) Change in inhibitory current onto the PC population of the rate model, during external stimulation of the FS/NFS population. Conditions at baseline and for the NFS/FS rate models correspond to Fig 4. A paradoxical reduction of inhibitory current onto the PC population, despite external stimulation of the inhibitory population, was observed in the FS model but not during baseline conditions or the NFS model, confirming the presence of an ISN regime. (TIF) [file pcbi.1009521.s005.tif]

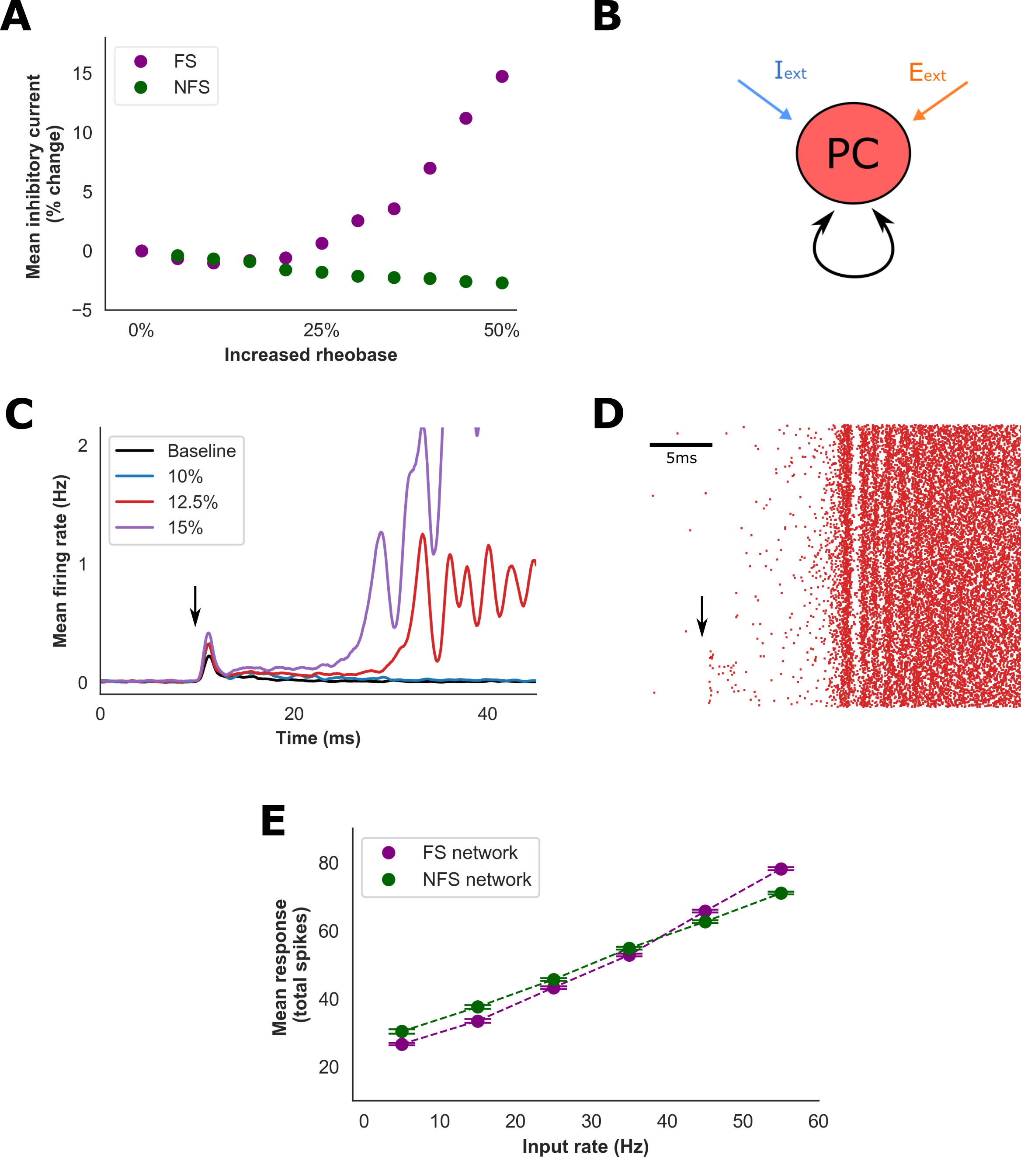

Supplement: S6 Fig — A) Change in total inhibitory current onto the PC population with increased FS rheobase. A reduction of inhibitory current is observed with increased FS rheobase before the network transitions to an ISN. Although increased NFS rheobase was associated with greater reductions of inhibitory current these changes did not produce an increase in PC gain (Fig 6). B) To investigate the impact of reductions of inhibitory input upon PC gain, a network of PC neurons (“the PC network”) was developed with external stimulation that reproduced the mean inhibitory and excitatory current of the original three-population network. C) Mean population response of the PC network in response to a brief stimulus (black arrow) after a reduction of external inhibition (coloured traces). A reduction greater than ~10% was sufficient to generate unrestrained excitatory activity, demonstrated by the raster plot for one simulation in D. E) Total number of spikes generated by the FS and NFS interneuron networks in response to excitatory stimuli shown in Fig 8. We observed small differences in total spikes elicited by each interneuron population, and at lower input rate fluctuations more spikes were elicited by the NFS interneuron network. (TIF) [file pcbi.1009521.s006.tif]

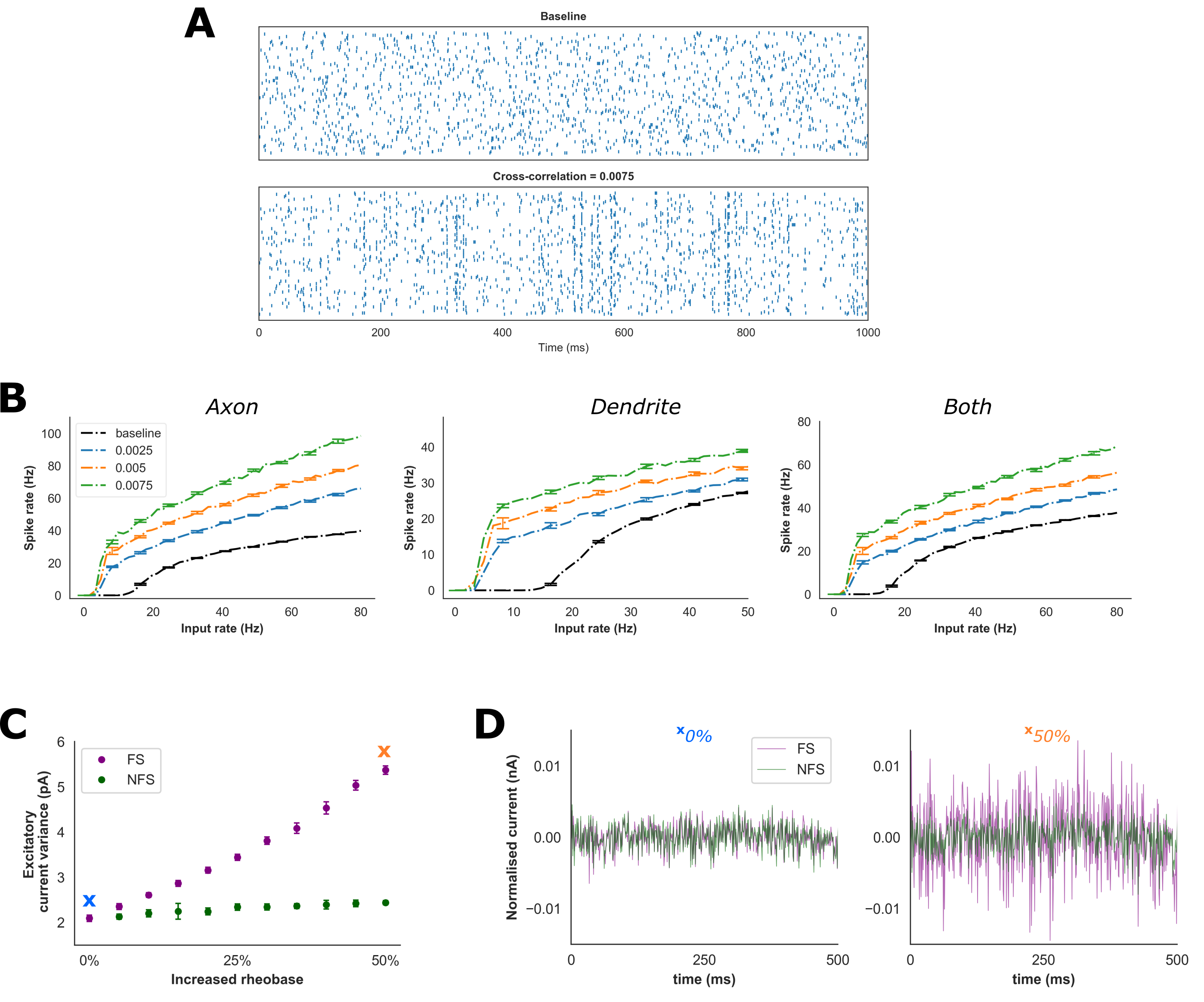

Supplement: S7 Fig — A) Raster plots for spike trains generated using a Poisson-process (baseline) and after introducing pair-wise spike correlations. Both spike trains have identical mean firing rate. B) Input-output relationship of PC neurons in response to generated spike trains of increasing rate (x-axis) and pair-wise spike correlation (colours). Inputs were applied to either the soma, dendrite or distributed equally across both compartments (“Both”). The presence of stronger spike correlations enhanced the excitability of the PC neuron model: at an input rate that elicits a spike frequency of 10Hz for uncorrelated inputs, a spike cross-correlation of 0.0025 elicited a frequency of 29.5Hz (P < 0.001, Welch’s t-test). Error bars denote s.e.m. C) Variance of excitatory synaptic input derived from internally generated excitatory network activity during conditions of increased FS and NFS rheobase. C) Greater variance with increased FS rheobase is appreciated visually from the traces of mean-normalised excitatory current input during baseline (left) and increased FS/NFS rheobase (right, purple/green traces respectively). (TIF) [file pcbi.1009521.s007.tif]

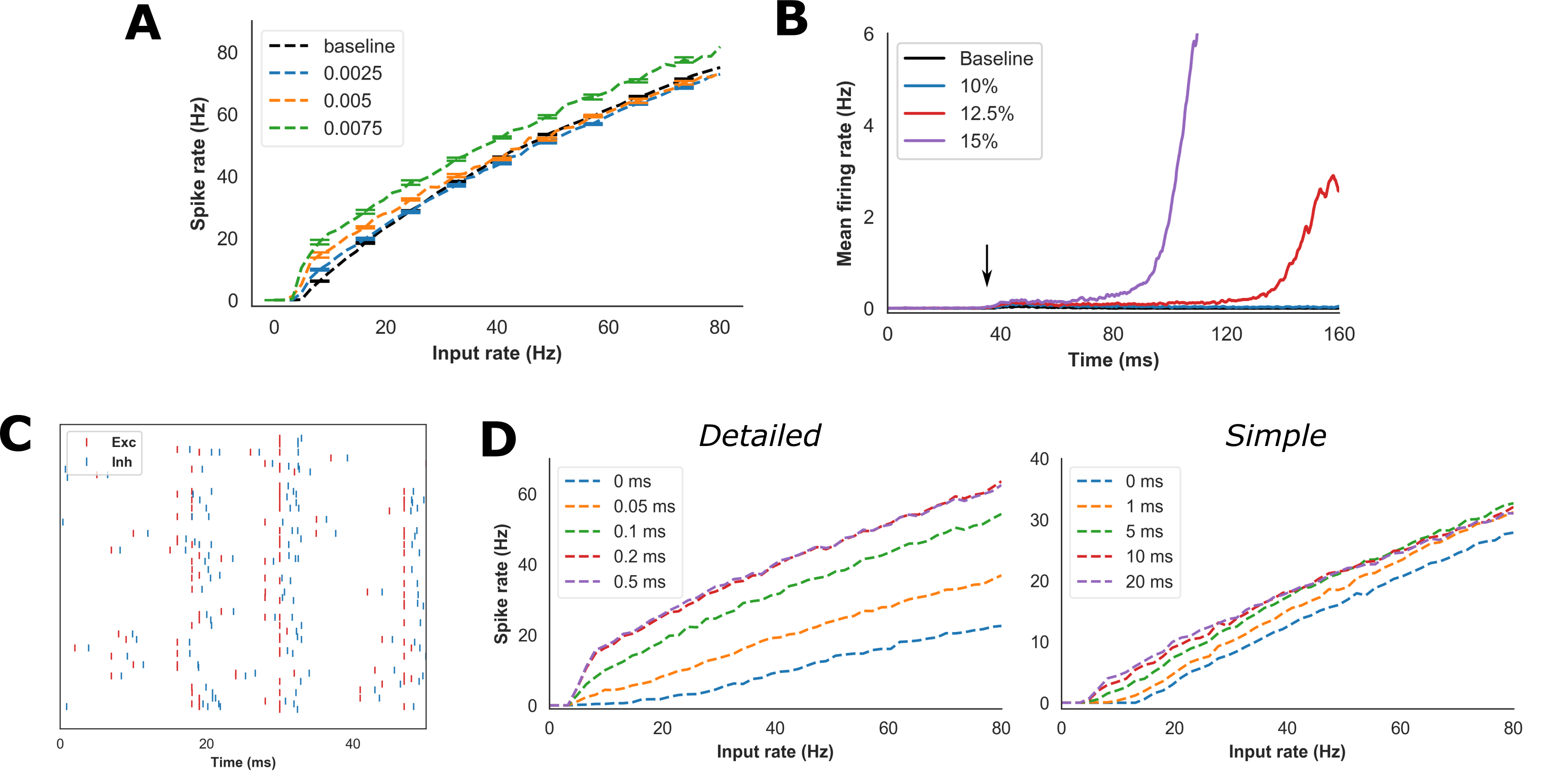

Supplement: S8 Fig — A) Input-output relationship of the single compartment Izhikevich PC model in response inputs of increasing rate and spike correlation (colour coded). Compared to the original PC model (S7 Fig), correlated synaptic inputs had less impact upon the excitability of the Izhikevich PC model: at a rate that elicits a spike frequency of 10Hz for uncorrelated inputs, a spike cross-correlation of 0.0025 elicited a frequency of only 13Hz (P < 0.001, Welch’s t test). B) Mean population response of the Izhikevich PC network in response to a brief stimulus (arrow) after a reduction of external inhibition. Similar to the PC network in S6 Fig, reduced inhibition generated unrestrained excitatory activity consistent with enhanced PC-to-PC synaptic gain. C) To explore the timing of inhibition, both the detailed and Izhikevich (simple) PC models were stimulated with identical excitatory and inhibitory spike trains, and a delay introduced to the inhibitory inputs of increasing duration. Here, inhibitory spikes have a mean delay of 2.0 ms compared to excitatory spikes. Input-firing rate relationship of the detailed (B) and Izhikevich (C) PC models with lengthening of inhibitory delays (color-coded). We found that the detailed model was sensitive to small delays of inhibitory input under 1ms: a delay of 0.5 ms increased the mean spike rate from 5 Hz to 34 Hz (P < 0.001, Welch’s t-test). Furthermore, delays greater than 0.5 ms had insignificant further impact on excitability. Although the Izhikevich PC model was also sensitive to inhibitory delays, the magnitude of the change in excitability was considerably smaller: a delay of 10 ms increased the mean spike rate from 5 to 11 Hz (P < 0.001). Changes to excitability occurred across a wider range of values, and delays greater than 10 ms did not exert a significant additional impact on excitability. (TIF) [file pcbi.1009521.s008.tif]

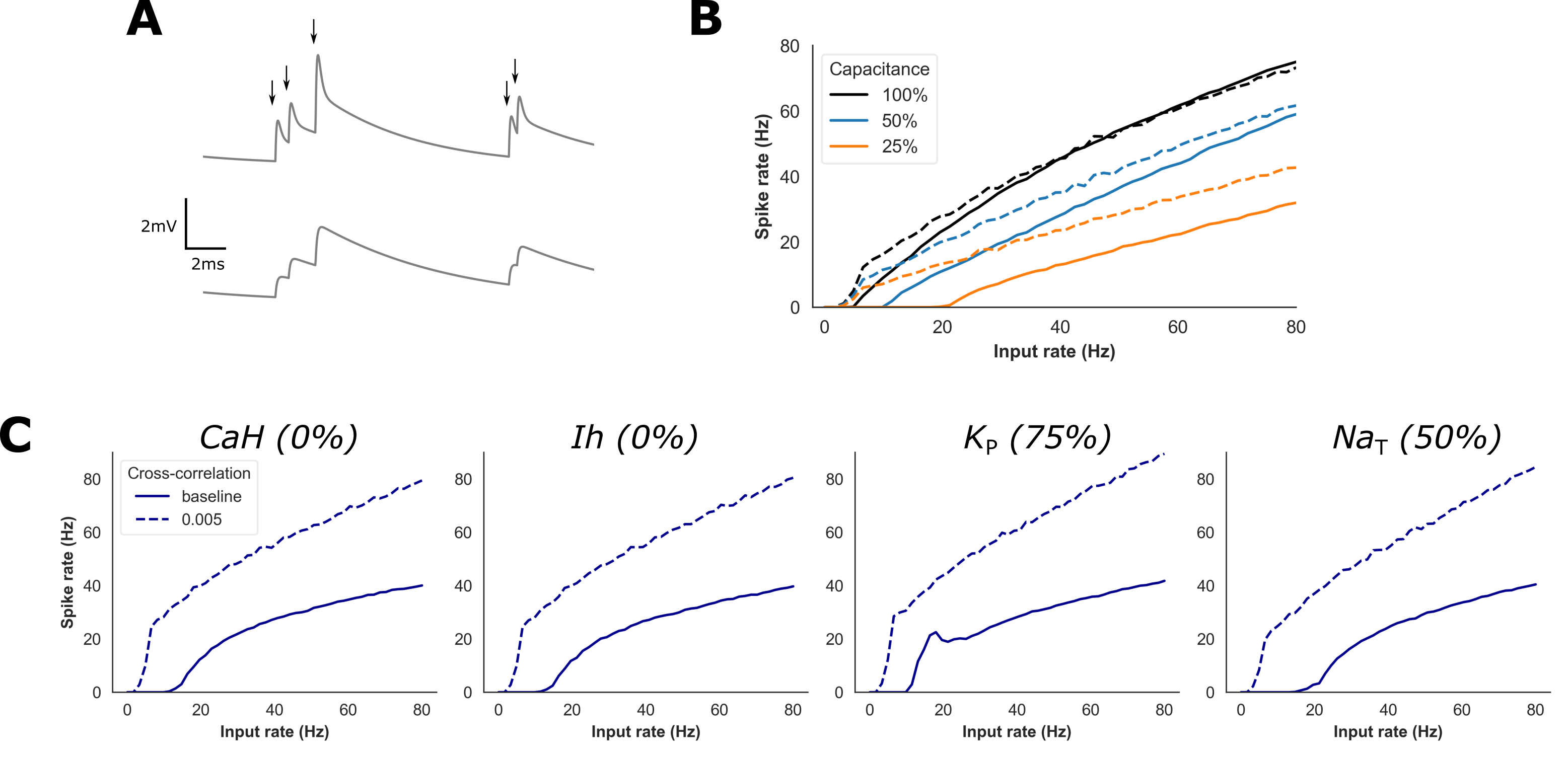

Supplement: S9 Fig — A) Time-voltage trace of the original (top) and Izhikevich (bottom) PC model in response to excitatory synaptic inputs (arrows). Membrane voltage decays more rapidly in the original PC model. B) Mean input-firing rate relationship of the Izhikevich model in response uncorrelated (solid line) and correlated (pair-wise cross-correlation of 0.005, dashed line) input after reducing membrane capacitance to 50 and 25% (blue and orange traces, respectively). Reducing the membrane time constant of the Izhikevich model by reducing membrane capacitance increases the sensitivity of the model to correlated input. For instance, the mean spike threshold at 25% capacitance decreased from 19.6 to 3.3Hz for inputs with a pairwise cross-correlation of 0.005, compared to 4.9 to 3.3Hz at 100% capacitance. C)The impact of other mechanisms for conferring sensitivity to correlated inputs in the detailed PC model was also explored by reducing the conductance of each ion channel mechanism, and then re-calculating the input-output relationship in response to uncorrelated (solid line) and correlated (dashed line) input. No mechanisms could abolish sensitivity to input correlations, further supporting the importance of the rate of decay of synaptic input. Here, enhanced sensitivity to correlated inputs was clearly retained despite complete removal of the CaH and Ih channels. Reductions of KP and NaT conductance below 75% and 50% respectively produced a loss of sustained firing. (TIF) [file pcbi.1009521.s009.tif]
