# Supplementary material for: State transitions through inhibitory interneurons in a cortical network model
Source: PLoS Comput Biol. 2021 Oct 15;17(10):e1009521. doi: 10.1371/journal.pcbi.1009521 (PMC8550371; doi:10.1371/journal.pcbi.1009521)
Supplement: S1 Table — AP: action potential; BAP: back-propagating action potential; AHP: after-hyperpolarization potential; ISI: inter-spike interval; ms: millisecond; mV: millivolt. (DOCX) [file pcbi.1009521.s010.docx]

| **Stimulus (compartment)** | **Feature (compartment)** | **Mean ± SD (units)** |
| --- | --- | --- |
| *#1*  1.9nA for 5ms (soma) | *AP peak*  *(soma)* | *25 ± 10 (mV)* |
|  | *AHP depth*  *(soma)* | *-65 ± 4 (mV)* |
|  | *AP half-width*  *(soma)* | *2 ± 0.5 (ms)* |
|  | *Spike count*  *(soma)* | *1 ± 0.001* |
|  | *Time to first spike*  *(soma)* | *2.8 ± 0.2 (ms)* |
|  | *BAP amplitude*  *(dendrite)* | *36 ± 9.3 (mV)* |
|  | *Spike count*  *(dendrite)* | *1 ± 0.001* |
|  | *Time to first spike*  *(dendrite)* | *6 ± 0.2 (ms)* |
| *#2*  1.9nA for 5ms (soma)  0.2nA for 5ms (dendrite, at offset of somatic stimulus) | *AP peak*  *(soma)* | *25 ± 10 (mV)* |
|  | *AP half-width*  *(soma)* | *2 ± 0.5 (ms)* |
|  | *Mean ISI*  *(soma)* | *10 ± 0.9 (ms)* |
|  | *Spike count*  *(soma)* | *3 ± 0.001* |
|  | *Ca^2+^ spike peak*  *(dendrite)* | *6.7 ± 2.5 (mV)* |
|  | *Ca^2+^ spike width*  *(dendrite)* | *37.4 ± 1.3 (ms)* |
| *#3*  0.5nA for 500ms (soma) | *Spike count*  *(soma & dendrite)* | *5 ± 1* |
| *#4*  0.5nA for 500ms (soma) | *Spike count*  *(soma & dendrite)* | *10 ± 1* |
|  | *Resting membrane potential*  *(dendrite)* | *-70 ± 5 (mV)* |
